# Evaluation of Atmospheric Preprocessing Methods and Chlorophyll Algorithms for Sentinel-2 Imagery in Coastal Waters

**DOI:** 10.3390/rs17203503

**Published:** 2025-10-21

**Authors:** Tori Wolters, Naomi E. Detenbeck, Steven Rego, Matthew Freeman

**Affiliations:** 1 Oak Ridge Institute for Science and Education, Oak Ridge, TN 37830, USA; 2 Atlantic Coastal Environmental Sciences Division, United States Environmental Protection Agency (US EPA), Narragansett, RI 02882, USA; 3 Applied Research Associates, Raleigh, NC 27615, USA

**Keywords:** algorithms, atmospheric correction, chlorophyll, remote sensing, estuary

## Abstract

Cyanobacterial blooms have been increasingly detected in estuaries and freshwater tidal rivers. To enhance detailed monitoring, an efficient approach to detecting algal blooms through remote sensing is needed to focus more detailed monitoring focused on cyanobacteria. In this study, we compared different remote sensing processing methods to determine an efficient approach to mapping chlorophyll-a. Using a subset of paired chlorophyll-a observations with Sentinel-2 imagery (2015–2022), with sites located in the Chesapeake Bay and Indian River selected along gradients of salinity, turbidity, and trophic status, we compared the combined performance of two different atmospheric processing methods (Acolite, Polymer) and a suite of empirical (band ratio, spectral shape indices) and machine learning algorithms for chlorophyll-a prediction. Acolite outperformed Polymer, resulting in 176 observation points, compared to 106 observation points from Polymer, and a greater range in chlorophyll-a values (0–74 μg/L from Acolite compared to 0–36 μg/L from Polymer), although Polymer showed more responsiveness at lower chlorophyll-a levels. Two algorithms performed best in predicting chlorophyll-a, as well as trophic state and HABs risk classes: the machine learning mixture density network (MDN) approach and the one band-ratio approach (Mishra).

## 1. Introduction

Cyanobacterial blooms have been detected with increasing frequency in subtropical estuaries [1], freshwater tidal systems [2,3], and brackish water sites along the coast of southern California [4]. Puget Sound may potentially be affected by freshwater-derived microcystins from upstream lakes and rivers, which are accumulating in Puget Sound shellfish to levels of concern and thus could pose a health risk to tribes collecting local shellfish for food [5,6]. Research on inland lakes has developed relationships between chlorophyll-a (Chl-a) concentrations and the probability of occurrence of cyanobacteria or cyanotoxins [7], and similar relationships are needed for coastal systems. In spite of the increasing detection of cyanobacterial blooms, Chl-a, cyanobacterial counts, and cyanotoxins are not routinely monitored across large spatial scales in coastal rivers and estuaries. Therefore, an efficient approach to detecting blooms through remote sensing is needed to focus on more detailed monitoring and to assess the environmental conditions (nutrients, temperature, flow regimes) associated with these blooms. Information on historical and near real-time frequency, occurrence, and magnitude of cyanobacterial blooms in lakes and reservoirs is being developed by the National Aeronautics and Space Administration (NASA)-funded Environmental Protection Agency (EPA) Cyanobacteria Assessment Network project (CyAN) based on remote sensing data from the European Space Agency (ESA) Envisat MEdium Resolution Imaging Spectrometer (MERIS) and Copernicus Programme Sentinel-3 satellites [8].

The detection of algal pigments via remote sensing requires data from multispectral sensors. However, data from some multispectral sensors such as MERIS (2002–2012) and Sentinel-3 (2016–present), with a 300 m spatial resolution, may be too coarse for smaller estuarine systems [9]. Data are available from the joint NASA/USGS Landsat 8 (30 m, 16-day interval, 2013–present) and the Copernicus Programme’s Sentinel-2 (10–60 m, 5–10-day interval, 2015–present) satellites at a finer spatial resolution. However, there are technical challenges involved in processing Sentinel-2 imagery for estimating Chl-a in coastal systems. Coastal waters are optically complex, with constituents such as colored dissolved organic matter (CDOM) and suspended solids that can interfere with the estimation of Chl-a [10]. Thus, Chl-a algorithms developed for the open ocean do not perform well in coastal waters. The detection of clouds and cloud shadows over water is also problematic for Sentinel-2, as the Sen2COR processing method developed by the European Space Agency (ESA) for Sentinel-2 was optimized for the detection of clouds over land, not water [11]. Cloud coverage is an issue in remotely sensed imagery, with the global average for cloud cover at 66%, and therefore must be dealt with in imagery processing [12].

Because of its importance in monitoring multiple aspects of water quality, such as nutrients and trophic state, Chl-a is frequently monitored or derived from remote sensing [12]. While the methods used to obtain Chl-a estimates vary across studies [13], it is a common parameter to analyze. Diganta et al. [12] stated that the process for optically complex coastal systems requires advanced approaches to overcome potential limitations of the different methods of deriving Chl-a in those ecosystems. Chl-a retrieval can fall into four categories: empirical (EM), semi-empirical/analytical (SA), hybrid algorithms, or data-driven machine learning (ML) and artificial intelligence (AI) [14]. The empirical algorithm category can be further split into band-ratio (R) or spectral shape indices (SS). Each category has limitations and advantages depending on the imagery, atmospheric correction methods, post-processing, and ecosystem of interest. The empirical algorithms are typically straightforward and provide accurate results; however, they can be limited in transferability when they are developed using a specific water body. Empirical algorithms originally developed for the open ocean, where interferences from nonalgal particulates and colored dissolved organic matter are low, were derived from maximum band ratios of blue and green remote sensing reflectance [15]. As the concentration of interfering TSS and CDOM increases in coastal waters, these constituents increase the spectral slope of reflectances in the blue-green range and lead to an overestimation of chlorophyll [15]. More recent ratio-based approaches have relied on red and near-infrared (NIR) bands with lower absorption coefficients for non-algal particulates and CDOM [16–19]. These ratio-based approaches perform well for areas with high algal biomass but have a low signal-to-noise ratio in low biomass areas [20]. Semi-empirical/analytical models can be accurate in some instances [21,22] but require accurate estimates of optical water parameters, and the results depend on the AC method used [23,24]. The ML/AI algorithms can offer precise results and are especially useful in processing big data. ML/AI algorithms are limited by their need for large training datasets, which may lead to uncertainty in estimates for some concentrations of Chl-a, or high cloud coverage areas [14]. Hybrid methods can be optimized for different optical water types, but their application is more complex and time-consuming [25].

Both atmospheric correction programs and prediction algorithms contribute errors to the final estimates of chlorophyll-a from remote-sensing images. As a result of molecular and aerosol scattering in the atmosphere, the contribution of atmospheric path radiance in the visible spectrum is greater than water-leaving radiance by 80–90% [26], and these effects must be removed to estimate the surface reflectance of the water itself. Atmospheric corrections rely on the calculation of atmospheric variables using a radiative transfer model, the estimation of the predominant contributors to absorption and scattering (aerosols and water vapor), and the subsequent conversion of Top-Of-Atmosphere (TOA) to Bottom-Of-Atmosphere (BOA) reflectance [27]. Sun-glint [28,29] and adjacency effects from nearby land [30] create additional complications for atmospheric corrections. The application of chlorophyll algorithms contributes additional sources of uncertainty, including the spatial and temporal heterogeneity of in situ chlorophyll, introducing errors in matching in situ with satellite measurements [31,32], the varying accuracy and precision of different in situ and laboratory methods for estimating chlorophyll [33], linearity assumptions for some empirical approaches to develop algorithms [34], and potential interferences from nonalgal particulates and colored dissolved organic matter.

To meet the need for improved monitoring of Chl-a in coastal systems, we chose (1) to evaluate a new cloud and cloud-shadow masking program for Sentinel-2, S2Cloudless (https://developers.google.com/earth-engine/datasets/catalog/COPERNICUS_S2_CLOUD_PROBABILITY accessed on 15 October 2025), (2) to compare three different atmospheric correction (AC) algorithms for Sentinel-2, (3) to evaluate the performance of existing Chl-a algorithms using observations from estuaries and freshwater tidal rivers paired with Sentinel-2 images [35], and (4) to compare our results with the historical performance of algorithms tested with a limited geographic scope and/or limited Optical Water Type (OWT). We compared the performance of three AC programs: Acolite v.20210114.0 [36], Polymer v.4.17 [37]), and the Sensor Invariant Atmospheric Correction v.2.3.5 (SIAC) program [38]. We chose to test the performance of Acolite and Polymer atmospheric correction programs because of their prevalent use, history of successful use in other water bodies and optical water types, ease of use, and level of documentation available at the time this project was started. We included SIAC in our original testing, although it was relatively untested because it would have provided the opportunity to generate atmospheric corrections that were consistent across Sentinel and Landsat images, as well as generating uncertainty estimates [39], but the need to access ancillary datasets from the MODIS MCD43 product created a bottleneck in processing, which extended processing time beyond a practical limit. SeaDAS [40] was not included for comparison due to installation difficulties, given EPA’s computing environment. C2RCC [41] was not tested because it is included in the Sentinel-3 Toolbox, but we needed to apply it to Sentinel-2. OC-SMART [42]was only released in 2020 and had relatively little mention in the literature at the time we started this project.

Our overall goal was to determine the best performing method for the better detection of Chl-a from remotely sensed data across a range of salinity (0–37 psu), turbidity (0–327 NTU), and Chl-a (0–211.6 μg/L) levels.

## 2. Materials and Methods

### 2.1. Test Point Selection

We extracted matched datasets of reflectances from Sentinel-2 imagery and Chl-a observations from the paired dataset compiled by Rego et al. [33]. To get a representative subsample encompassing the diverse coastal environment, we selected Chl-a observations randomly along gradients of Chl-a level, turbidity, and salinity. The parent dataset spanned significant ranges of these parameters: 0–36 ppt salinity, nd–332 NTU turbidity, 0.1–100 μg Chl-a/L [33]. The Chl-a observations were obtained from various monitoring programs, and all sources included QA/QC flags that were used in filtering out samples that did not meet US EPA QA/QC standards (Table 1, Figure 1).

Some of the highest Chl-a values were filtered out by quality assurance and quality control (QAQC) processes; thus, we added targeted high values of Chl-a associated with known estuary blooms to extend the range of Chl-a values represented. Optical water classes described by fuzzy cluster analysis of the parent database had similar spectral signatures to those described as typical of CDOM-dominated waters but lacked classes characteristic of chlorophyll-dominated waters without optical interferences [33]. Given the external source of chlorophyll data from monitoring programs with disparate goals, we were not able to integrate chlorophyll values across optical depth; the parent dataset included samples collected at depths of 0.13–3 m, i.e., not integrated across the visible depth [33]. Chlorophyll values in the parent database represented a range of methods. Most values (90.2%) were from calibrated in vivo measurements from buoys or towed samplers. The in vitro sample analyses generally followed US EPA Methods 445.0 and 447.0 [48,49], and only phaeophytin-corrected values were included in the database.

### 2.2. Cloud and Cloud Shadow Masking Process

The original cloud mask incorporated into Level-2C (L2C) Sentinel imagery was generated by ESA using Sen2COR. However, Sen2COR was designed for use on terrestrial images and does not work as well over water [11]. The improved S2Cloudless indicator was only incorporated into L2C Sentinel imagery starting in January 2022 (https://developers.google.com/earth-engine/datasets/catalog/COPERNICUS_S2_CLOUD_PROBABILITY (accessed on 15 October 2025)). Thus, we used the Google Earth Engine (GEE) S2cloudless app (https://showcase.earthengine.app/view/s2-sr-browser-s2cloudless-nb (accessed on 15 October 2025)) to generate a list of potential tile dates for estuaries with less than 10% cloud cover based on the S2cloudless indicator. We incorporated cloud and cloud shadow masks into Sentinel L1 downloads using a modified GEE script (https://developers.google.com/earth-engine/tutorials/community/sentinel-2-s2cloudless (accessed on 15 October 2025)). The latter requires specifying a cloud probability threshold, which is set to 0.5 as the default. Developers recommend plotting a histogram of cloud probabilities for images and choosing thresholds that best discriminate between peaks corresponding to clear versus cloudy conditions. In addition to peaks associated with clear and cloudy conditions, we found a third peak that could be discriminated using a threshold of 0.2, and which we surmised was associated with a different form of clouds, so we used the lowest threshold in generating cloud and cloud shadow masks.

The GEE script applies a two-stage method to identify cloud shadows. The first stage applies a water mask before identifying potential cloud shadow regions, as there is difficulty in distinguishing between cloud shadows and water pixels, as both exhibit low near-infrared (NIR) values. That step had to be skipped because we were dealing with water images. The second stage (which we retained) assumes cloud shadows will be cast in a direction opposite to the solar azimuth, with a distance based on expected cloud height.

### 2.3. Atmospheric Corrections and Post-Processing Steps

We processed the downloaded Sentinel-2 images using the EPA High Performing Computer Cluster (HPCC) and compared the effectiveness of two atmospheric correction programs, Acolite and Polymer, by processing all images through both. We initially included SIAC processing in our comparisons, but the SIAC method was not feasible, given the number of tiles we had to process. Both Acolite and Polymer allow users to define an area of interest for clipping the original tiles. Individual polygons were created for each estuary based on Estuary Data Mapper coverages [50] and then buffered by 30 m to account for fluctuating water levels. The bounding boxes coordinates were exported as points to be included as inputs in the Acolite and Polymer programs. Acolite and Polymer outputs were Network Common Data Form (NetCDF) files. Post-processing steps were performed to ensure NetCDF images were Climate and Forecasting (CF) compliant [51], checking data type, variable naming convention, and missing data, as well as defining metadata with variable descriptions and spatial and temporal extent of the data. The CF-compliant NetCDF files were then downloaded and imported to ArcGIS Pro version 2.9 (©ESRI, Redlands, CA, USA).

Additional image processing was needed once we pulled them into ArcGIS Pro. Using ArcGIS Pro ModelBuilder, we developed a workflow to batch process the images. We read in the images using an iterator along with the tool “make netCDF feature layer.” As a result of the post-processing methods, the images were oriented upside down; part of the ModelBuilder workflow included converting the images to rasters temporarily, then using the “flip” tool to orient correctly and converting back to feature classes. Finally, we clipped the images and performed a spatial join with the subsample of matched points.

### 2.4. QAQC Filtering

Following the join of the Sentinel-2 images and the matched buffered test points, the outputs went through the QAQC program in R [52]. The estuarine Chl-a sample points were buffered by a 30 m radius for the extraction of surface reflectance data. Buffered sample points that intersected shorelines or buffered roads (representing bridges or causeways) were excluded from the matching dataset to avoid adjacency effects. The QAQC filtering was done on three wavelengths: 443 (aerosol), 493 (blue), and 566 (green). First, we calculated the mean, standard deviation (SD), and confidence interval of ±1.5 SD, then filtered out points that fell outside that range. Following the filtering, we recalculated the mean and standard deviation and found the coefficient of variation (CV). We also calculated the median and the number of pixels after filtering. This process was repeated for each of the three wavelengths. Next, we found the minimum number of pixels and calculated the median CV across all three wavelengths. Finally, we kept the values if the minimum number of pixels was greater than 16 and the median CV fell below the CV threshold. We compared results using three different CV thresholds from the literature: 0.15, 0.4 [53], and 0.2 [54]. We evaluated the results of the different CV thresholds based on the number of points that remained after filtering at each level.

### 2.5. Chl-a Algorithm Comparison

Next, we proceeded with our comparisons of the atmospheric correction programs and various Chl-a calculations. We evaluated the results from Acolite and Polymer by comparing the number of points that were filtered out, the number of negative values, and the number of outliers from each program. We compared several Chl-a algorithms from the literature: MDN [55], normalized difference Chl-a index (NDCI) [56], Moses3b and Moses3b 740 [19], Mishra [57], Gons and Gons 740 [18], maximum Chl-a index (MCI) [58], and maximum peak height (MPH) [59]. These algorithms fell under a subset of the categories of Chl-a algorithms: MDN is an ML/AI algorithm; the other algorithms we compared all fall under the empirical category and can be broken down into spectral shape indices (MCI, MPH, and NDCI) or band ratio algorithms (Gons, Mishra, Moses) [14]. We set up another program in R with the equations for each Chl-a metric and compared the results in scatterplots.

Using the methods outlined in Salls et al. [58], we performed a statistical analysis of the Chl-a estimates from the algorithms compared with the observed Chl-a. We developed a program in R to perform the statistical analysis. We performed a model II regression using the lmodel2 package [60] in R and used the Ranged Major Axis output in our analysis. We included statistics on the slope, intercept, r^2^, confidence intervals, mean absolute multiplicative error (MAE), mean absolute percentage error (MAPE), and multiplicative mean bias (MB).

$$\text{MAE}=10^{\wedge \left(\frac{\text{sum}\left|\text{log}10\left(\text{predicted}\right)-\text{log}10\left(\text{observed}\right)\right|}{\text{n}}\right)}$$

$$\text{MAPE}=100*\frac{\text{sum}\left(\frac{\left|\text{predicted}-\text{observed}\right|}{\text{predicted}}\right)}{\text{n}}$$

$$\text{MB}=10^{\wedge \left(\frac{\text{sum}\left(\text{log}10\left(\text{predicted}\right)-\text{log}10\left(\text{observed}\right)\right)}{\text{n}}\right)}$$

We performed this analysis twice: once with all predicted Chl-a values and once with negative predicted Chl-a values removed. We evaluated our results based on the regressions and associated statistics for each Chl-a algorithm.

We also evaluated the ability of different Chl-a algorithms to predict trophic classes and to identify threshold exceedances for risk associated with harmful algal blooms. We used the trophic class thresholds [61] (low: <5 μg/L, medium: >5 μg/L–<20 μg/L, high: >20 μg/L–<60 μg/L, and hypereutrophic: >60 μg/L) and cyanobacteria exceedance thresholds (low: <23 μg/L, medium >23 μg/L–<68 μg/L, and high: >68 μg/L) [7] to find the classification rates for each Chl-a metric. We found the frequency of values for each threshold level, then used confusion matrices to calculate the accuracy and miscalculation rates.

## 3. Results

Atmospheric correction outputs from Acolite resulted in the loss of fewer points during the QAQC filtering process and fewer negative reflectance values compared with Polymer. The number of valid matched observations that remained after atmospheric corrections was 542 for Acolite but only 219 for Polymer. After QAQC filtering, 426 valid observations remained for Acolite, and only 148 remained for Polymer. Additional visual filtering was done to remove observations affected by clouds or artifacts (e.g., islands, boat wakes), leaving 172 observations for Acolite and only 106 for Polymer. Some observations yielded negative predicted Chl-a values, and these were removed before final regressions. See Tables 2 and 3 for the final number of observations included in regression analyses. Observed Chl-a range for Acolite (after filtering) was 0–74 μg·L^−1^, while the range for Polymer after filtering was 1.6–36 μg·L^−1^. Based on a combination of (1) the degree of data loss during atmospheric processing, (2) overcorrection by AC programs leading to negative Chl-a predictions, and (3) precision and accuracy of predicted Chl-a, we determined that overall, Acolite was performing better than Polymer for our systems (Tables 2 and 3). All algorithms except MDN and NDCI produced higher bias for Chl-a estimates when using Polymer-processed data than when using Acolite-processed data. The variance explained was consistently greater for Acolite-processed data in spite of a lower number of observations. However, the precision was better for the Polymer results.

We found that the CV threshold of 0.4 produced the best results for Acolite-processed data across all Chl-a algorithms, with r^2^ values increasing and MAPE values decreasing as the CV threshold was increased from 0.1 to 0.4. (Results based on application of different CV thresholds are provided in Supplemental Figures S1–S15 and Supplemental Tables S1–S4). Based on the number of points retained after QAQC filtering with CV = 0.4, r^2^ values, MAPE, and bias values, MDN [55] (Figure 2), Mishra [57] (Figure 3), and Gons 740 [18] (Figure 4) were the best performing Chl-a algorithms (Table 2). However, the Gons 740 results were heavily influenced by the largest Chl-a value (Figure 4). Several of the algorithms yielded negative Chl-a predictions, resulting in more restricted ranges for calibration curves (Gons, Moses3b, Moses3b (740), and MCI). Spectral shape indices had high error rates (46,734–infinite) (Table 2).

Our comparison of misclassification rates and accuracy based on the trophic class threshold and cyanobacteria threshold is reported in Table 4, also with negative Chl-a predictions removed. The misclassification rate was calculated as false positives plus false negatives, divided by the total predictions for each Chl-a algorithm. Similarly, accuracy was calculated as the true positives plus true negatives, divided by the total predictions for each algorithm. The total number of predictions for these calculations was the same as that used in the algorithm comparison. Except for Moses3b740, all the Chl-a algorithms were moderately to highly accurate at discriminating pixels across the range of cyanobacteria thresholds (80–100%). Gons, Gons740, MDN, and Mishra had consistently high accuracies (>92%). Success in classifying trophic class categories was more variable across algorithms, with moderately high accuracies for MDN (>82%). The greatest variability in accuracy was in the lower trophic class thresholds (Chl-a < 5, and >5–<20 μg L^−1^) for most band-ratio algorithms.

With the exception of Pahlevan’s work [27,62], most previous evaluations of the performance of ACs and Chl-a algorithms with Sentinel have been limited in geographic scope and/or OWT (Table 5). Fit statistics for many of these efforts are not directly comparable to ours, as they performed regressions on log-transformed Chl-a, while we did not. For those systems described by regressions without log-transforms, MAPE was 24%, as compared to our values of 58–104% for top-performing combinations. Reported bias values in the literature (non-transformed) were relatively low at −0.6 to 0.5, but these studies evaluated limited OWTs. For Sentinel-3, Neil et al. [10] was able to compare the performance of multiple algorithm and AC combinations using global data (both inland and coastal) and concluded that the best combinations depended on OWT, suggesting that multiple methods should be used.

## 4. Discussion

### 4.1. Performance of Atmospheric Correction Algorithms

Although Acolite showed better performance overall with our dataset, Acolite showed a limited ability to discriminate among Chl-a levels below 5–20 μg chl/L (depending on the algorithm) as compared to higher values (Figures 2–4), and Polymer showed better responsiveness at lower values but more variability at higher Chl-a/turbidity, resulting in the loss of data (Supplemental Figures S1–S15, Supplemental Tables S1–S3). The accuracy of aquatic reflectance retrievals from Sentinel-2 imagery varies among wavelengths, AC algorithms, inland versus coastal water bodies, and OWT [37–39,41,74]. For two French coastal regions, Bui et al. [75] found similar performance of several atmospheric correction processors with low scatter and bias: NASA-AC, C2RCC, Polymer, ACOLITE, and OC-SMART (especially OC-SMART), but poorer performance for Sen2COR (default ESA processor for Sentinel-2) and iCOR. Pahloven et al. performed a more comprehensive assessment, considering seven different OWTs; OWTs 1 and 2 (clearer waters) are commonly found in the coastal waters and/or oligotrophic lakes, whereas OWT3 is attributed to moderately eutrophic waters [62]. Lakes or coastal estuaries with various degrees of phytoplankton blooms are represented by OWTs 4, 5, and 6. OWT7 represents systems with significant suspended sediments. The second Atmospheric Correction Intercomparison eXercise (ACIX-II) has evaluated eight different state-of-the-art AC, including Acolite and Polymer, using data from the Ocean Color component of the Aerosol Robotic Network (AERONET-OC; [54]) for coastal systems, as well as radiometric data from the Community Validation Database (CVD) for mainly inland water bodies. Overall, for both inland and coastal waters, the iCOR AC performed well for OWT 3–7 across visible bands but poorly for OWT 1 and 2, while C2X performed well for 443, 490, and 664 nm for OWT 1–4 and 6. For most wavelengths in the visible range (443–664 nm), Acolite performed relatively poorly for OWT 1–4, with the exception of excellent performance at 664 nm for OWT4, but performed moderately well to excellent for OWT 5–7. Acolite performance was best across the ACs for OWT 7 (turbid waters). For 560–664 nm, Polymer performed well for OWT 1–2 but poorly for other OWTs. These results may explain the better performance we observed for Chl-a algorithms in oligotrophic waters using the Polymer AC and the better performance of algorithms in predicting Chl-a in eutrophic waters using Acolite.

Other promising AC methods or improvements to existing AC methods have been proposed, but have been subjected to more limited testing [29,39,76,77]. We initially processed Sentinel-2 images using the newer SIAC AC process, which operates within a probabilistic (Bayesian) framework and has been reported to work well across all wavelengths with minimal bias [38]. However, the process was much slower than for Acolite and Polymer, and it was not feasible for processing large numbers of tiles.

ACIX-II data have also been used to evaluate the accuracy of a subset of Chl-a algorithms using results from the different AC processes, but in situ Chl-a measurements were only available for inland waters, so evaluations for coastal systems could only be based on estimated Chl-a from in situ radiometric instruments (Chl-a^r^). AC performance was only compared using MDN [55] and OCx [15] for coastal systems. For the estimation of Chl-a^r^ in inland waters, the combination of the OCx algorithm with CDX AC performed best (error = 67.4%, bias = 10.9%), but MDN with Acolite AC was almost as precise, with greater bias (error = 77.1%, bias = 33.1%). For estimation of Chl-a^r^ in coastal waters, the OCx algorithm with SeaDAS and OC-SMART ACs worked best. Applying either MDN or OCx with the Polymer AC produced major biases (MDN bias = −30.5%, OCx bias = −38.9%) as compared to lesser biases for Acolite (MDN bias = −10.8%, OCx bias = 15.2%). Our observed prediction errors and multiplicative bias for MDN with Acolite were even lower than those observed in the ACIX-II study (MAPE = 55.3%, bias = 2.5 μg·L^−1^), even though we were comparing predicted Chl-a with measured Chl-a rather than with Chl-a^r^, thus incorporating more sources of error.

### 4.2. Performance Compared to Original Algorithm Calibrations

We found that the Gons 740 algorithm was one of the top three best-performing (r^2^ = 0.72, sterr = 0.62); this algorithm replaces band 783 with band 740. Gons et al. [18] calibrated and validated two forms of the MERIS algorithm using sets of data from three years for calibration and three separate years for validation. Switching from the original wavelength at 672 to 665 nm resulted in slightly less accurate Chl-a predictions. This is partially due to a change in the absorption coefficient, which accounts for most of the inaccuracies from the Gons algorithm [56]. An updated 2008 version of the Gons algorithm did not perform well in waters with less than 5 mg m^−3^ but did work on a wide range of concentrations above that [78]. They reported r^2^ > 0.89 and sterr = 0.035. Mishra and Mishra [56] stated that algorithms that use wavelengths over 750 nm may not be as accurate, because those wavelength absorption coefficients reported in the literature are not as reliable. Additionally, atmospheric corrections are not as reliable for remote sensing reflectance (*R*_rs_) values at those higher bands. This could account for differences in the performance of the original Gons and the Gons 740 algorithms. We also used the updated version, using the 665 nm band, which could account for lower accuracy in our results. Another potential limitation was found by Salama et al. [79], who reported that standard algorithms (specifically Gons) performed poorly in optically complex waters.

Moses et al. [19] developed a three-band NIR-edge algorithm and achieved a MAE of 4.71 mg·m^−3^, as compared to our values of 1.02. Their algorithm worked well on a wide range of Chl-a concentrations. They compared their algorithms on several water bodies. The Moses algorithms (Moses3b and Moses3b 740) did not perform well on our dataset, resulting in r^2^ of 0.14 and 0.48, respectively. The Moses3b algorithm also uses band 783, while the Moses3b 740 replaces that with band 740. Once again, the lack of reliable absorption coefficients and *R*_rs_ values for the longer wavelengths could account for that [56].

In another study, Mishra et al. used NDCI-derived calibrations to predict Chl-a and achieved r^2^ = 0.98, although they did state that the results were best with a nonlinear fit [79]. The Mishra algorithm was one of the best performing (r^2^ = 0.64). We performed linear regressions, which could explain the difference in our results from Mishra et al. [57], although our results showed no evidence of nonlinearity.

Unlike other investigators, we found that none of the spectral shape indices performed well in our coastal systems after applying atmospheric corrections. Salls et al. [58] compared NDCI and MCI and found that MCI performed better for inland lakes. They also compared different reflectance types: Top-of-Atmosphere, Rayleigh-corrected, and *R*_rs_, and found the best performance of MCI was with Top-of-Atmosphere reflectance values, which could explain the difference in our results. Mishra and Mishra [56] found NDCI-predicted Chl-a with 12% bias over a range of water types and regions. In their study comparing multiple algorithms with NDCI, they found that NDCI performed the best with an r^2^ = 0.90, STE = 2.11 mg·m^−3^, and *p* < 0.0001 for coastal and estuarine waters. Matthews [59] developed the MPH algorithm, which resulted in an r^2^ of 0.58 and an error of 33.7% for cyanobacteria-dominant coastal and inland waters. This algorithm was developed using Top-of-Atmosphere reflectance values to avoid the need for atmospheric corrections. This could explain the differences between our results.

Pahlevan et al. [55] developed MDN to estimate Chl-a and compared it to other available algorithms. They reported that MDN performed better, resulting in a MAPE of 24.0, using Sentinel-2. We also found that MDN performed the best of all the algorithms we compared, resulting in an r^2^ of 0.80; however, our analysis resulted in an MAPE of 80.

### 4.3. Comparison of Sentinel-2 with Performance of Sentinel-3 in Coastal Systems

There may be tradeoffs when deciding whether to use Sentinel-2 or Sentinel-3 in coastal systems. We chose Sentinel-2 for the finer resolution and more frequent overpasses that allowed for greater matches with the observed dataset. Salama et al. [80] reported that there was greater uncertainty in Chl-a estimates (using Gons algorithm) from Sentinel-2 than from Sentinel-3, but the finer scale resolution of Sentinel-2 is still an advantage. Similarly, Caballero et al. [81] stated that both Sentinel-2 and Sentinel-3 achieved similar bloom patterns, but Sentinel-2 was more useful for small-scale bloom visualization. Another study found that both Sentinel-2 and Sentinel-3 performed well, but there was variation depending on the optical complexity of the site and the Chl-a algorithm used (they compared 2-, 3-, and 4-band algorithms) [65]. They also found that the two-band algorithms outperformed the three-band algorithms for both Sentinel-2 and Sentinel-3. Staehr et al. [82] also studied the use of Sentinel-3 in coastal systems, reporting that it was useful for getting the full spatial scale of the estuaries they studied; however, they found that Sentinel-3 overestimated Chl-a in shallow waters where benthic vegetation could interfere, and underestimated in inner estuaries that had shallow Chl-a-rich waters. Other studies also reported accurate Chl-a estimates using Sentinel-3 [10,74]. Overall, Sentinel-2 and Sentinel-3 seem to perform similarly, with some slight variation caused by several factors; therefore, the spatial scale of the site of interest is likely most important when deciding which Sentinel satellite to employ for Chl-a estimations.

### 4.4. Performance of Sentinel-2 for Coastal Versus Inland Waters

For ease of application, the AC and Chl-a algorithm methods selected for transitional waters, such as freshwater tidal rivers and estuaries, should be consistent across water types and salinity gradients. For this reason, we developed our methods across salinity gradients. Lock et al. [83] studied brackish lakes in Australia and found that Chl-a estimates varied by lake and year, reporting some overestimation but no significant relationships. They concluded that remote sensing products need fine-tuning based on individual sites, although they did acknowledge that the algorithms they compared may not always be accurate in shallower lakes such as their study site. Conversely, Salls et al. [59] used Sentinel-2 to estimate Chl-a in lakes and reported that it was effective for lakes across the United States. Pahlevan et al. [55] reported similar findings in their development of the MDN algorithm using various water sources globally, including lakes, reservoirs, and rivers. Both Salls and Pahlevan also used a range of trophic levels in their studies and still reported that Sentinel-2 was able to accurately predict Chl-a using various algorithms.

### 4.5. Practical Considerations in Implementation

For the implementation of our methods, some changes will be needed. Since we began this project, the USGS Bulk Download Application has been disabled, and the use of Google Earth Engine has been restricted for some Federal agencies in the United States. We now recommend using ESA Copernicus Browser [84] to download Sentinel-2 imagery. The Copernicus Browser has many of the same features, including setting an area of interest, cloud cover thresholds, and selecting between Sentinel missions.

USGS has recently released preprocessed Sentinel-2 data as a dynamic data product with a 20-m pixel resolution [85], applying the Acolite atmospheric correction. Also available is a script for on-the-fly use of the data, rather than downloading the whole tile. One limitation is that, at least so far, no improved cloud/cloud shadow masking is available. The CloudS2Mask package [86] has recently become available for improved cloud/cloud shadow masking of Sentinel L1C images, but downloading the Level 1 tiles will be necessary to incorporate this step.

For operational use, managers will need to consider a tradeoff between hybrid approaches that apply the best combination of ACs and Chl-a algorithms for each OWT and a single approach that is more streamlined but which may vary in precision over different OWTs. Our results suggest that application of either of our top two algorithms with Acolite preprocessing will do an excellent job in classifying images with respect to thresholds associated with HABs and moderately well (Mishra) or extremely well (MDN) in distinguishing among trophic categories.

## 5. Conclusions

Our goal was to determine the best methods for processing remotely sensed data to obtain chlorophyll estimates across a range of salinity, turbidity, and Chl-a levels for estuaries and coastal rivers. We compared both atmospheric correction methods and Chl-a algorithms. We found that Acolite AC performed better overall, while Polymer was more responsive at lower Chl-a values but resulted in a loss of data at higher ranges. Additionally, we found that MDN, Mishra, and Gons740 were the best-performing Chl-a algorithms across the range of Chl-a, salinity, and turbidity of our sites. Ultimately, although there are trade-offs to consider when implementing these methods, our results show great promise in an efficient approach to using remotely sensed data to predict Chl-a in coastal systems.

## Supplementary Material

Supplement1

**Supplementary Materials:** The following supporting information can be downloaded at: https://www.mdpi.com/article/10.3390/rs17203503/s1, Figure S1. Scatterplot comparing observed Chl-a and results from Gons [18] algorithm with the CV threshold at 0.4 (40%), and the corresponding statistical results. Data from Acolite atmospheric preprocessor.; Figure S2. Scatterplot comparing observed Chl-a and results from Moses3b [19] algorithm with the CV threshold at 0.4 (40%), and the corresponding statistical results. Data from Acolite atmospheric preprocessor.; Figure S3. Scatterplot comparing observed Chl-a and results from Moses3b 740 [19] algorithm with the CV threshold at 0.4 (40%), and the corresponding statistical results. Data from Acolite atmospheric preprocessor.; Figure S4. Scatterplot comparing observed Chl-a and results from MPH [59] algorithm with the CV threshold at 0.4 (40%), and the corresponding statistical results. Data from Acolite atmospheric preprocessor.; Figure S5. Scatterplot comparing observed Chl-a and results from MCI [58] algorithm with the CV threshold at 0.4 (40%), and the corresponding statistical results. Data from Acolite atmospheric preprocessor.; Figure S6. Scatterplot comparing observed Chl-a and results from NDCI [56] algorithm with the CV threshold at 0.4 (40%), and the corresponding statistical results. Data from Acolite atmospheric preprocessor.; Figure S7. Scatterplot comparing observed Chl-a and results from Gons [18] algorithm with the CV threshold at 0.4 (40%), and the corresponding statistical results. Data from Polymer atmospheric preprocessor.; Figure S8. Scatterplot comparing observed Chl-a and results from Gons 740 [18] algorithm with the CV threshold at 0.4 (40%), and the corresponding statistical results. Data from Polymer atmospheric preprocessor.; Figure S9. Scatterplot comparing observed Chl-a and results from MDN [55] algorithm with the CV threshold at 0.4 (40%), and the corresponding statistical results. Data from Polymer atmospheric preprocessor.; Figure S10. Scatterplot comparing observed Chl-a and results from Mishra [58] algorithm with the CV threshold at 0.4 (40%), and the corresponding statistical results. Data from Polymer atmospheric preprocessor.; Figure S11. Scatterplot comparing observed Chl-a and results from Moses3b [19] algorithm with the CV threshold at 0.4 (40%), and the corresponding statistical results. Data from Polymer atmospheric preprocessor.; Figure S12. Scatterplot comparing observed chlorophyll-a and results from Moses3b 740 [19] algorithm with the CV threshold at 0.4 (40%), and the corresponding statistical results. Data from Polymer atmospheric preprocessor. Figure S13. Scatterplot comparing observed Chl-a and results from MPH [59] algorithm with the CV threshold at 0.4 (40%), and the corresponding statistical results. Data from Polymer atmospheric preprocessor.; Figure S14. Scatterplot comparing observed Chl-a and results from MCI [58] algorithm with the CV threshold at 0.4 (40%), and the corresponding statistical results. Data from Polymer atmospheric preprocessor.; Figure S15. Scatterplot comparing observed Chl-a and results from NDCI [56] algorithm with the CV threshold at 0.4 (40%), and the corresponding statistical results. Data from Polymer atmospheric preprocessor.; Tables S1–S4: Fitting statistics for regressions of observed versus estimated chlorophyll based on various algorithms.

## Figures and Tables

**Figure 1.**
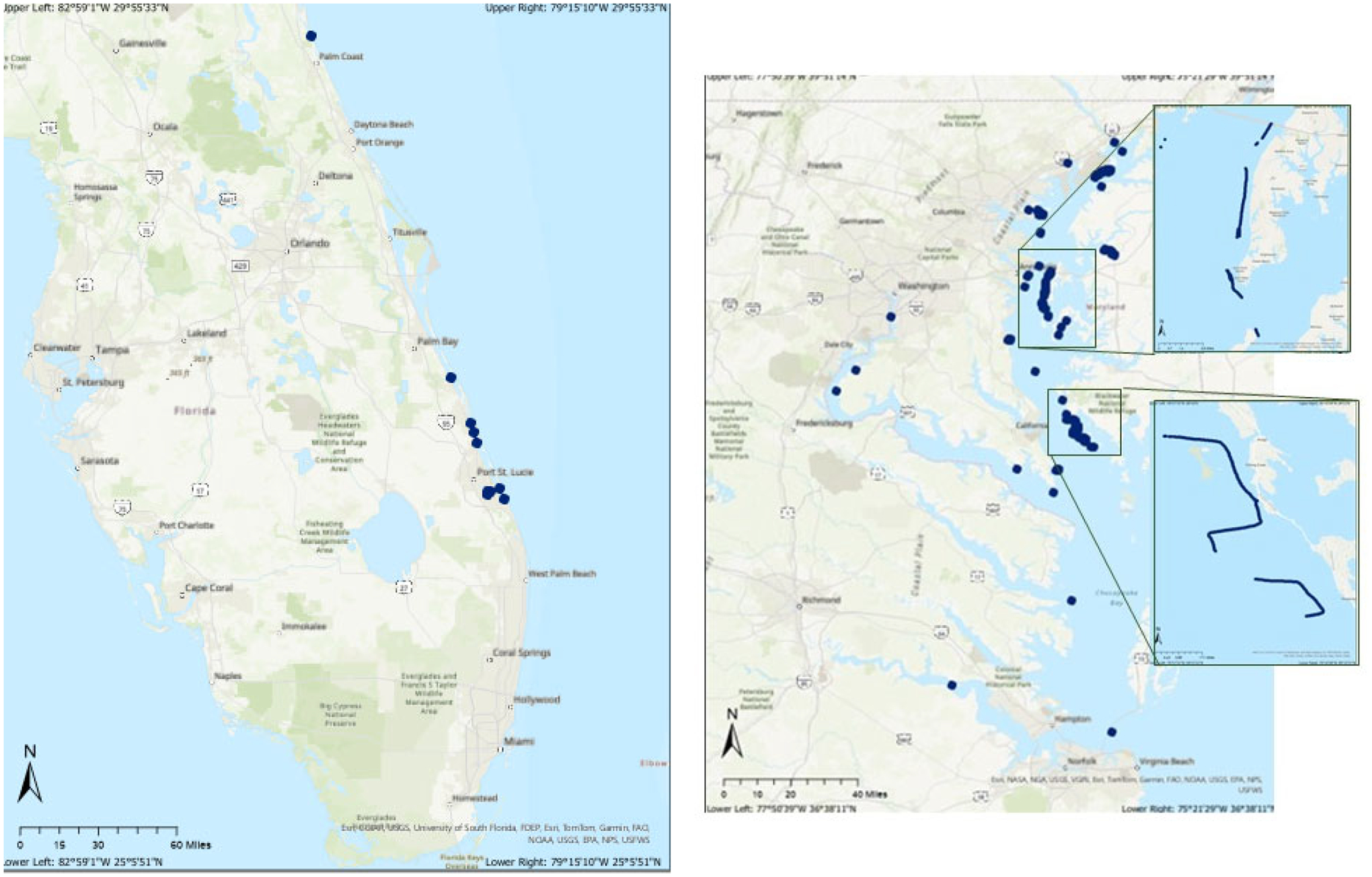
Map showing locations of the observation points–represented by blue points–that were used. Data obtained from sources in Table 1. Observation points came from Chesapeake Bay and Indian River Lagoon. Clusters of points represent transects of observation points, identified in the zoomed in boxes on the map. Basemaps: ESRI, NASA, USGS, University of South Florida, FDEP, ESRI, TomTom, Garmin, FAO, NOAA, USGS, EPA, NPS, USFWS [43]. ESRI, NASA, USGS, VGIN, ESRI, TomTom, Garmin, FAO, NOAS, USGS, EPA, NPS, USFWS [43].

**Figure 2.**
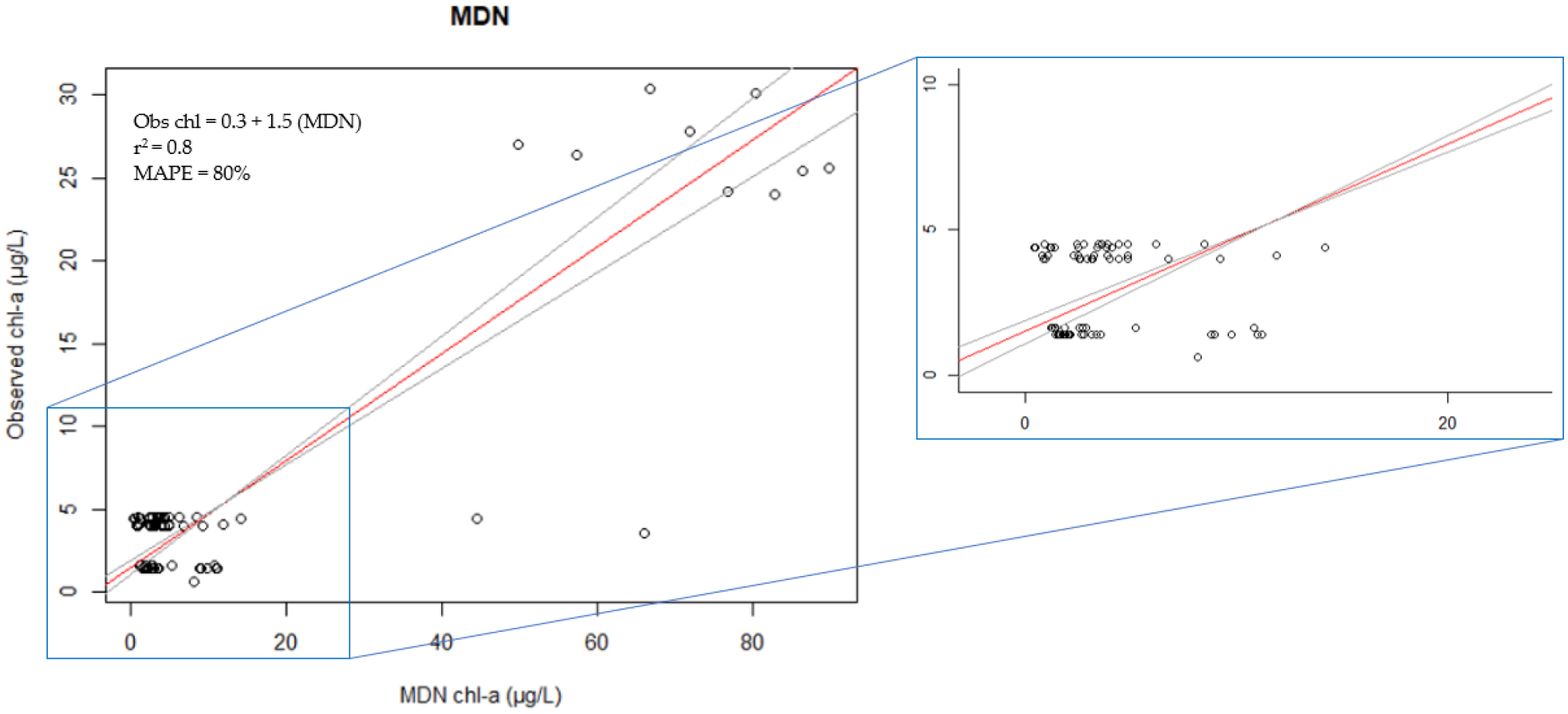
Scatterplot comparing observed chlorophyll-a and results from MDN [55] algorithm with the CV threshold at 0.4, and the corresponding statistical results.

**Figure 3.**
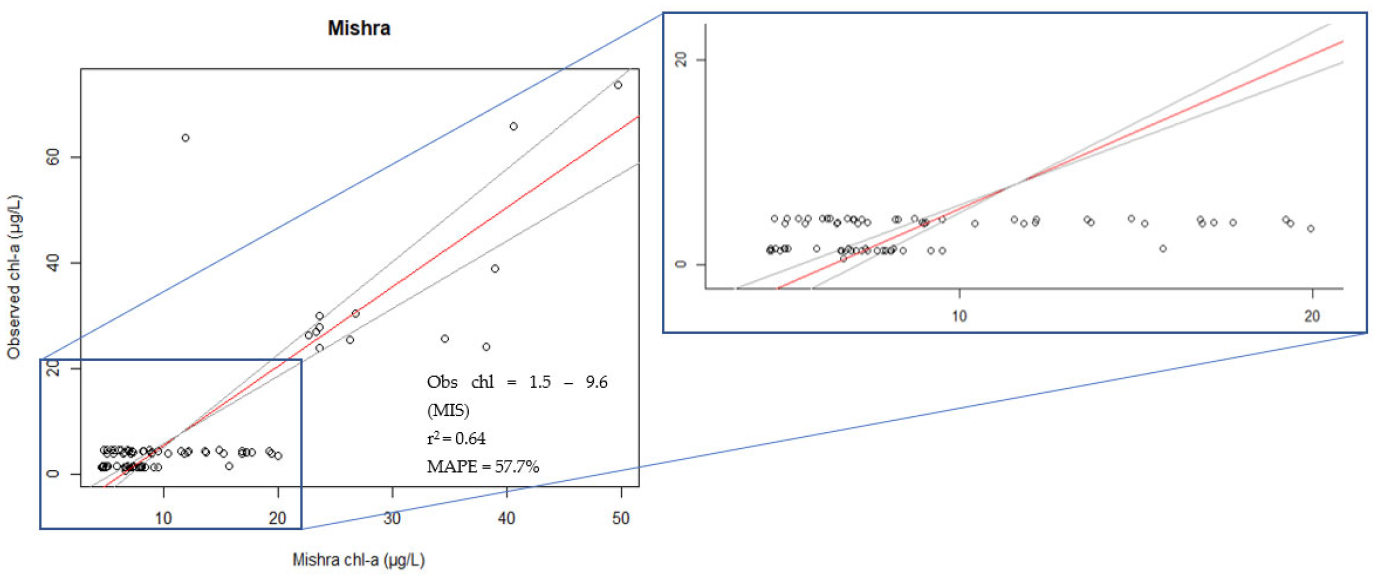
Scatterplot comparing observed chlorophyll-a and results from Mishra [57] algorithm with the CV threshold at 0.4 (40) and the corresponding statistical results.

**Figure 4.**
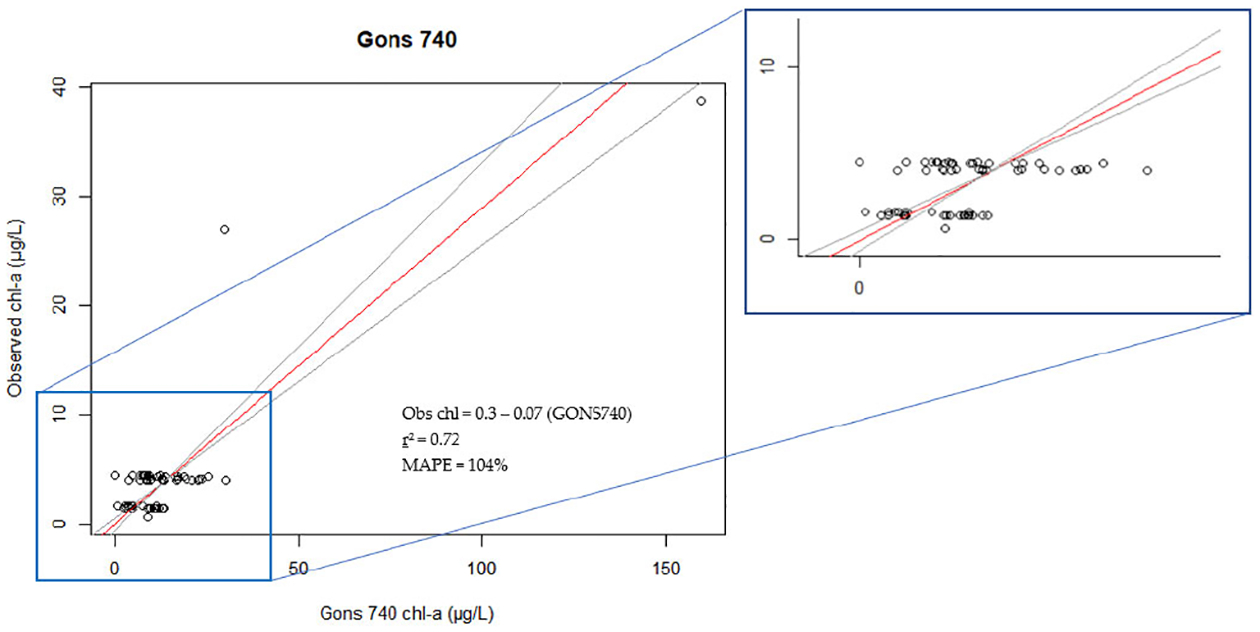
Scatterplot comparing observed chlorophyll-a and results from Gons 740 [19] algorithm with the CV threshold at 0.4 (40%) and the corresponding statistical results.

**Table 1.** Sources and information about observed Chl-a data used for sample points.

Data Source	Name	Partners	Data Information	QA/QC
[44]	Indian River Lagoon Observatory Network of Environmental Sensors	Operated by the Indian River Observatory at Harbor Branch Oceanographic Institute	10 stations, with real-time monitoring Web-based interface for data download	Yes Both sensors and data have QA/QC processes
[45]	FerryMon	North Carolina DOT operated ferries Data is collected and downloaded via UNC Chapel Hill Marine Laboratory.	Real-time data can be downloaded directly. Past data must be requested.	Yes Data is checked using EPA QA/QC standards
[46]	Maryland “Eyes on the Bay”; Chesapeake Bay River Input Monitoring Program	Supported through MD DNR and USGS	Continuous monitoring—need station name, date range, and chosen parameter(s) to download	Yes
[47]	Chesapeake Bay Tidal Water Quality Monitoring	In partnership with Maryland, Virginia, and US EPA	100+ stations with continuous monitoring	Yes

**Table 2.** Chl-a algorithm statistical analysis comparison using values corrected with Acolite atmospheric preprocessor, with negative values removed. Bolded values indicate those that are statistically significant. CI = confidence interval, sterr = standard error, MAE_mult_ = multiplicative mean absolute error, MAPE = mean absolute percentage error, MB = multiplicative bias. Algorithm classification is as follows: EM = empirical, ML = machine learning; sub-categories of empirical algorithms: R = band ratio, LH = line height.

	Chlorophyll-a Algorithms								
Statistic	Gons	Gons740	MDN	Mishra	Moses3b	Moses3b740	MCI	MPH	NDCI
Algorithm classification	EM: R	EM: R	ML	EM: R	EM: R	EM: R	EM: LH	EM: LH	EM: LH
Reference	[18]	[18]	[55]	[57]	[19]	[19]	[58]	[59]	[56]
n	73	72	89	93	30	25	49	88	72
Intercept	0.005	−0.07	1.5	−9.6	1.4	1.3	−20	2.8	−2
Slope	0.3	0.3	0.3	1.5	0.1	0.05	22,409	1541	249
*p*-value	**0.001**	**0.001**	**0.001**	**0.001**	**0.012**	**0.001**	0.08	**0.001**	**0.001**
CI2.5_int	−0.7	−0.7	1.1	−13	−8	−0.3	77	1.6	−6.3
CI97.5_int	0.6	0.5	1.9	−7.1	2.5	2.1	−2.9	3.8	1.1
CI2.5_slop	0.2	0.3	0.3	1.3	0.04	0.03	8797	1228	181
CI97.5_slop	0.3	0.3	0.4	1.8	0.6	0.1	−54,985	1891	348
r^2^	0.64	0.72	0.80	0.64	0.13	0.4	0.04	0.5	0.36
Mean	3.7	3.7	5.4	7.7	3.3	3.1	8.3	8	9.2
Median	4.0	4.0	4.0	4.0	4.0	4.0	4.0	4.0	4.0
sterr	0.61	0.62	0.8	1.4	0.25	0.3	2.34	1.5	1.7
MAE	1.02	1.02	1.0	1.02	1.02	1.0	1.09	Infinite	1.01
MAPE	988.2	103.8	80	57.7	46.2	47.8	Infinite	Infinite	46,734
MB	9.2	9.2	3.1	6.8	7.6	2.4	0.001	0	0.36

**Table 3.** Chl-a algorithm statistical analysis comparison using values corrected with Polymer atmospheric preprocessor, with negative values removed. Bolded values are those that are statistically significant. CI = confidence interval, sterr = standard error, MAE_mult_ = multiplicative mean absolute error, MAPE = mean absolute percentage error, MB = multiplicative bias, NA = not available.

	Chlorophyll-a Algorithms								
Statistics	Gons	Gons740	MDN	Mishra	Moses3b	Moses3b 740	MCI	MPH	NDCI
Reference	[18]	[18]	[55]	[57]	[19]	[19]	[58]	[59]	[56]
n	33	33	92	91	67	83	49	90	11
Intercept	8.9	9.1	3	4.6	4.9	4.5	1.5	4.5	4.4
Slope	−0.3	−0.3	0.14	−0.002	−0.004	−0.003	183.1	−216.2	−1.4
*p*-value	0.1	0.1	**0.001**	**0.003**	**0.001**	**0.001**	0.42	**0.001**	0.24
CI2.5_int	6.3	6.3	2.9	4.2	4.6	4.3	NA	4.2	NA
CI97.5_int	−5.9	−5.8	3.1	6	5.3	5	NA	5	NA
CI2.5_slope	0.7	0.7	0.12	−0.006	−0.005	−0.004	NA	−3570.1	NA
CI97.5 slope	−0.1	−0.12	0.18	−0.001	−0.003	−0.002	NA	−1346.7	NA
r^2^	0.061	0.06	0.57	0.1	0.42	0.25	0.0002	0.17	0.065
Average	4.65	4.65	3.64	3.64	3.76	3.7	3.03	3.64	3.24
Med	5.1	5.1	3.3	3.3	3.3	3.4	3	3.4	2.8
sterr	0.2	0.2	0.12	0.11	0.14	0.13	0.1	0.12	0.31
MAE	1.0	1.0	1.0	1.0	1.0	1.0	1.1	1.1	1.0
MAPE	20	20	9708	67.6	63.2	77.3	infinite	infinite	13,119
MB	38.4	37.8	0.75	53.5	427.7	166.2	0.003	0.003	0.25

**Table 4.** Threshold classification rates and accuracy for different Chl-a algorithms, following Acolite processing, based on Chl-a results with negative values removed.

		Trophic Class Thresholds				Cyanobacteria Thresholds		
		<5 μg/L	>5–<20 μg/L	>20–<60 μg/L	>60 μg/L	<23 μg/L	>23–<68 μg/L	>68 μg/L
		Low	Medium	High	Hypereutrophic	Low	Medium	High
Gons	Accuracy	0.24	0.27	0.95	0.97	1	0.97	0.97
	Misclassification	0.76	0.73	0.05	0.03	0	0.03	0.03
Gons740	Accuracy	0.29	0.32	0.94	0.97	0.99	0.99	0.97
	Misclassification	0.71	0.68	0.06	0.03	0.01	0.1	0.03
MDN	Accuracy	0.84	0.82	0.92	0.95	0.98	0.9	0.92
	Misclassification	0.16	0.18	0.07	0.05	0.02	0.1	0.08
Mishra	Accuracy	0.24	0.21	0.98	0.95	0.95	0.95	1
	Misclassification	0.76	0.79	0.02	0.05	0.05	0.05	0
Moses3b	Accuracy	0.36	0.6	0.7	0.94	0.82	0.82	1
	Misclassification	0.64	0.4	0.3	0.06	0.18	0.18	0
Moses3b740	Accuracy	0.3	0.74	0.74	0.82	0.63	0.81	0.81
	Misclassification	0.7	0.26	0.26	0.18	0.37	0.19	0.19
MCI	Accuracy	0.86	1	0.96	0.9	0.86	0.88	0.98
	Misclassification	0.14	0	0.04	0.1	0.14	0.12	0.02
MPH	Accuracy	0.83	1	0.89	0.95	0.83	0.84	0.99
	Misclassification	0.17	0	0.11	0.05	0.17	0.16	0.01
NDCI	Accuracy	0.8	1	0.84	0.96	0.8	0.8	0.99
	Misclassification	0.2	0	0.16	0.04	0.2	0.2	0.01

**Table 5.** (**a1**). Previous tests of Chl-a algorithms with Sentinel-2 data in coastal systems compiled from the literature. AC = atmospheric correction: A = Acolite, P = Polymer, S = Sen2Cor, OC = OC-SMART, C2 = C2RCC, R = Raleigh-corrected, U = unspecified, EN = ENVI. Algorithm types: ML = machine learning, R = ratio, LH = line height, EM = empirical, SA = semi-analytical. Chlorophyll algorithms: MDN = mixture density network, OC = ocean color, NDCI = normalized difference chlorophyll index, 3BI = three-band index, SS = spectral shape, MCI = maximum chlorophyll index, PLS = partial least squares, WASI4 = Web Assembly System Interface, LGBM = light gradient boosting machine, RF = random forest, MLP = multilayer perceptron, GPR = Gaussian process regression, SVM = support vector machine. MAE = mean absolute error, APD = absolute percent difference, RPD = relative percent difference, OWT = optical water type. (**a2**). Performance of Chl-a algorithms with Sentinel-2 data in coastal systems. Statistics based on regressions with log transforms are shaded. Statistics: root mean square error (RMSE), root mean square logarithmic error (RMSLE), mean absolute percentage error (MAPE), bias, mean absolute error (MAE), absolute percent difference (APD), relative percent difference (RPD), and r^2^. Compilation of results reported in the literature. (**b1**). Previous tests of Chl-a algorithms with Sentinel-3 data in coastal systems compiled from the literature. AC = atmospheric correction: A = Acolite, P = Polymer, S = Sen2Cor, OC = OC-SMART, C2 = C2RCC, R = Raleigh-corrected, U = unspecified, EN = ENVI, MU = multi. Algorithm types: ML = machine learning, R = ratio, LH = line height, EM = empirical, SA = semi-analytical. Chlorophyll algorithms: MDN = mixture density network, OC = ocean color, NDCI = normalized difference chlorophyll index, 3BI = three-band index, SS = spectral shape, MCI = maximum chlorophyll index, PLS = partial least squares, WASI4 = Web Assembly System Interface, LGBM = light gradient boosting machine, RF = random forest, MLP = multilayer perceptron, GPR = Gaussian process regression, SVM = support vector machine. MAE = mean absolute error, APD = absolute percent difference, RPD = relative percent difference, OWT = optical water type. (**b2**). Performance of Chl-a algorithms with Sentinel-3 data in coastal systems. Statistics based on regressions with log transforms are shaded. Statistics: root mean square error (RMSE), root mean square logarithmic error (RMSLE), mean absolute percentage error (MAPE), bias, mean absolute error (MAE), absolute percent difference (APD), relative percent difference (RPD), and r^2^. Compilation of results reported in the literature.

a1										
	AC	Algorithm type (ML/R/LH/EM/SA	Chl-a algorithms compared		Sign. Band(s)		Best performing algorithm		Log-transformed?	*n*
Gulf of Maine, USA, estuaries, OWT = low turbidity										
1	P	ML	MDN						Y (bias, MAE)	145
2	A	ML	MDN						Y (bias, MAE)	121
3	S	ML	MDN						Y (bias, MAE)	71
4	OC	ML	MDN						Y (bias, MAE)	162
5	C2	ML	MDN				MDN/P		Y (bias, MAE)	148
6	P	R	OC3/OC4						Y (bias, MAE)	163
7	A	R	OC3/OC4						Y (bias, MAE)	135
8	S	R	OC3/OC4						Y (bias, MAE)	68
9	OC	R	OC3/OC4						Y (bias, MAE)	153
10	C2	R	OC3/OC4						Y (bias, MAE)	153
Mundaú-Manguaba Estuarine-Lagoon System, Brazil, OWT = moderate chl, TSS levels										
11		R	Morel and Prieur		705/665				N	14
Rio de la Plata Estuary, South America, OWT = high turbidity, eutrophic										
12	A	R	NDCI		665, 705					43
13	A	R	3BI [16])		665, 705, 740		three-band index (3BI [16])			43
14	A	R	IDrozd		665, 705, 560					38
	A	R	SS(665)		560, 705					43
Coastal area of Sema-rang, Indonesian estuaries, OWT = high turbidity, eutrophic										
15	U				490, 560, 665, 842					
Ha Long Bay of Vietnam										
16	S	R	OC-2 [15]		490, 555					
United States, mostly lakes with some estuaries, OWT = multiple but excluded high turbidity										
17	None				665, 705, 740		MCI		Y (bias, MAE)	44
18	R	LH	MCI [58]		665, 705, 740		MCI		Y (bias, MAE)	46
19	S				665, 705, 740		MCI		Y (bias, MAE)	49
20	None				665, 705				Y (bias, MAE)	49
21	R	LH	NDCI [56]		665, 705				Y (bias, MAE)	48
22	S				665, 705				Y (bias, MAE)	48
Sado Estuary, Portugal, OWT = low turbidity and chl										
23	A	EM	OC3 [15]		443, 490, 560				Y (except RPD, APD)	11
24	A	SA	two-band [19]		665, 705				Y (except RPD, APD)	11
25	A	SA	[18]		665, 705, 783				Y (except RPD, APD)	11
26	P	ML	C2RCC [63]		All				Y (except RPD, APD)	13
27	P	EM	OC3 [15]		443, 490, 560				Y (except RPD, APD)	13
28	C2	ML	C2RCC [63]		All				Y (except RPD, APD)	13
29	C2	EM	OC3 [15]		443, 490, 560				Y (except RPD, APD)	13
30	C2	SA	two-band [19]		665, 705				Y (except RPD, APD)	13
31	C2	SA	[18]		665, 705, 783		Gons w C2		Y (except RPD, APD)	13
Yura Estuary, Japan, OWT = low turbidity and chl										
32	EN	SA	SDA				SDA		Y	
Mar Piccolo, Italy, estuary										
33	A	R	PLS, OC3, WASI4				PLS			
34		R	OC3							
35		SA	WASI4							
South Korea, lakes and estuaries, low-high chl, low–moderate TSS										
36		ML	LGBM, RF, MLP, GPR, blending algorithm, SVM		704, 665, 739, 492, 560		LGBM			
China, Estonia, Germany, Japan, New Zealand, South Korea, USA, Vietnam, lakes, bays, estuaries										
37		ML	MDN						Y (bias, MAE)	
a2										
	n	RMSE	RMSLE	MAPE	Bias	MAE	APD	RPD	r^2^	Ref.
Gulf of Maine, USA, estuaries, OWT = low turbidity										
1	145	2.89	0.39	49%	1.27	2.02				[64]
2	121	8.14	0.59	118%	0.4	2.78				
3	71	4.67	0.53	130%	0.49	2.73				
4	162	4.71	0.52	74.60%	0.64	2.54				
5	148	5.15	0.56	67.40%	0.66	2.69				
6	163	3.1036	0.49	91%	0.51	2.43				
7	135	3.25	0.48	68.50%	0.56	2.45				
8	68	66.7	1.22	1167%	0.1	11.1				
9	153	17	0.73	204%	0.35	3.93				
10	153	11.12	0.76	162%	0.32	3.98				
Mundaú-Manguaba Estuarine-Lagoon System, Brazil, OWT = moderate chl, TSS levels										
11	14	6.39							0.53	[65]
Rio de la Plata Estuary, South America, OWT = high turbidity, eutrophic										
12	43	6.4				3.7			0.96	[66]
13	43	4.9				4.8			0.98	
14	38	17.8							0.77	
	43	7.2				4.7			0.96	
Coastal area of Sema-rang, Indonesian estuaries, OWT = high turbidity, eutrophic										
15		2.47		24%	−0.63				0.45	[67]
Ha Long Bay of Vietnam										
16		2.8							0.81	[68]
United States, mostly lakes with some estuaries, OWT = multiple but excluded high turbidity										
17	44			100.9	1.15	2.08			0.5	[58]
18	46			100.1	1.05	2.22			0.49	
19	49			307.3	1.17	2.47			0.47	
20	49			220.1	1.52	2.41			0.46	
21	48			323.4	1.51	3.12			0.31	
22	48			318.4	1.53	2.68			0.02	
Sado Estuary, Portugal, OWT = low turbidity and chl										
23	11	0.515			0.461		224.3	224.3	0.217	[69]
24	11	1.044			1.018		1116	1116	0.0003	
25	11	0.327			−0.115		61.6	−1.59	0.0004	
26	13	0.503			0.543		221.2	216.1	0.365	
27	13	0.603			0.543		309.7	304.1	0.294	
28	13	0.714			0.242		281.1	243.6	0.223	
29	13	0.885			0.717		749.4	733.9	0.259	
30	13	0.765			0.754		493	493	0.608	
31	13	0.565			−0.527		67	−67	0.625	
Yura Estuary, Japan, OWT = low turbidity and chl										
32		0.162							0.76	[70]
Mar Piccolo, Italy, estuary										
33									0.115	[71]
34										
35										
South Korea, lakes and estuaries, low-high chl, low-moderate TSS										
36		15.15			0.15	9.49			0.75	[72]
China, Estonia, Germany, Japan, New Zealand, South Korea, USA, Vietnam, lakes, bays, estuaries										
37		30.3	0.61	24	0.99	1.27				[55]
b1										
	AC	Algorithm type	Chl-a algorithms compared	Sign. Band(s)	Best performing algorithm		Log-transformed?		n	
		Northern Persian Gulf semi-enclosed marginal sea, OWT = eutrophic								
1	iCOR	R	OC4M	442, 554, 667	NN, OC5		Y		31	
2	iCOR	R	OC5				Y		20	
3	iCOR	ML	NN	all			Y		18	
World-wide inland + coastal. OWT = all										
4	None, in situ reflectance	MU	48	708, 665	Two-band [73]		Y			
5	None, in situ reflectance	MU	48		Multi-algorithm x OWT scheme		Y			
b2										
	n	RMSE	RMSLE	MAPE	Bias	MAE	APD	RPD	r^2^	Ref.
Northern Persian Gulf semi-enclosed marginal sea, OWT = eutrophic										
1	31	0.31			0.23		87.9	75.41	0.30	[74]
2	20	0.15			0.13		52.97	40.08	0.56	
3	18	0.23			0.2		56.88	47.99	0.62	
World-wide inland + coastal. OWT = all										
2		0.256			0.057	0.188			0.78	[10]
3		0.25			0.02	0.18			0.79	[10]

## Data Availability

The original data presented in the study are openly available in data.gov at https://catalog.data.gov/dataset/figures-1-3-and-example-code-for-observed-chlorophyll-vs-sentinel-2-estimated-chlorophyll (accessed 15 October 2025).

## Highlights

### What are the main findings?

- The Acolite atmospheric correction program outperforms Polymer.
- Chlorophyll algorithms performed best at a CV threshold of 0.4, and MDN and Mishra algorithms achieved the best results.

### What is the implication of the main findings?

- Satellite imagery (Sentinel-2 vs. Sentinel-3), atmospheric correction programs, and chlorophyll algorithms can be used in various combinations depending on the area of interest, spatial and temporal scales, and the optical water types.
- Tradeoffs must be considered when deciding to use a hybrid approach for varying optical water types vs. a streamlined approach, which may result in varying precision across optical water types.
