# Supplementary material for: Evaluation of Atmospheric Preprocessing Methods and Chlorophyll Algorithms for Sentinel-2 Imagery in Coastal Waters
Source: Remote Sens (Basel). Author manuscript; Available in PMC 2026 Oct 21. (PMC12774331; doi:10.3390/rs17203503)
Supplement: Supplementary file 1 [file NIHMS2123289-supplement-Supplement1.pdf]

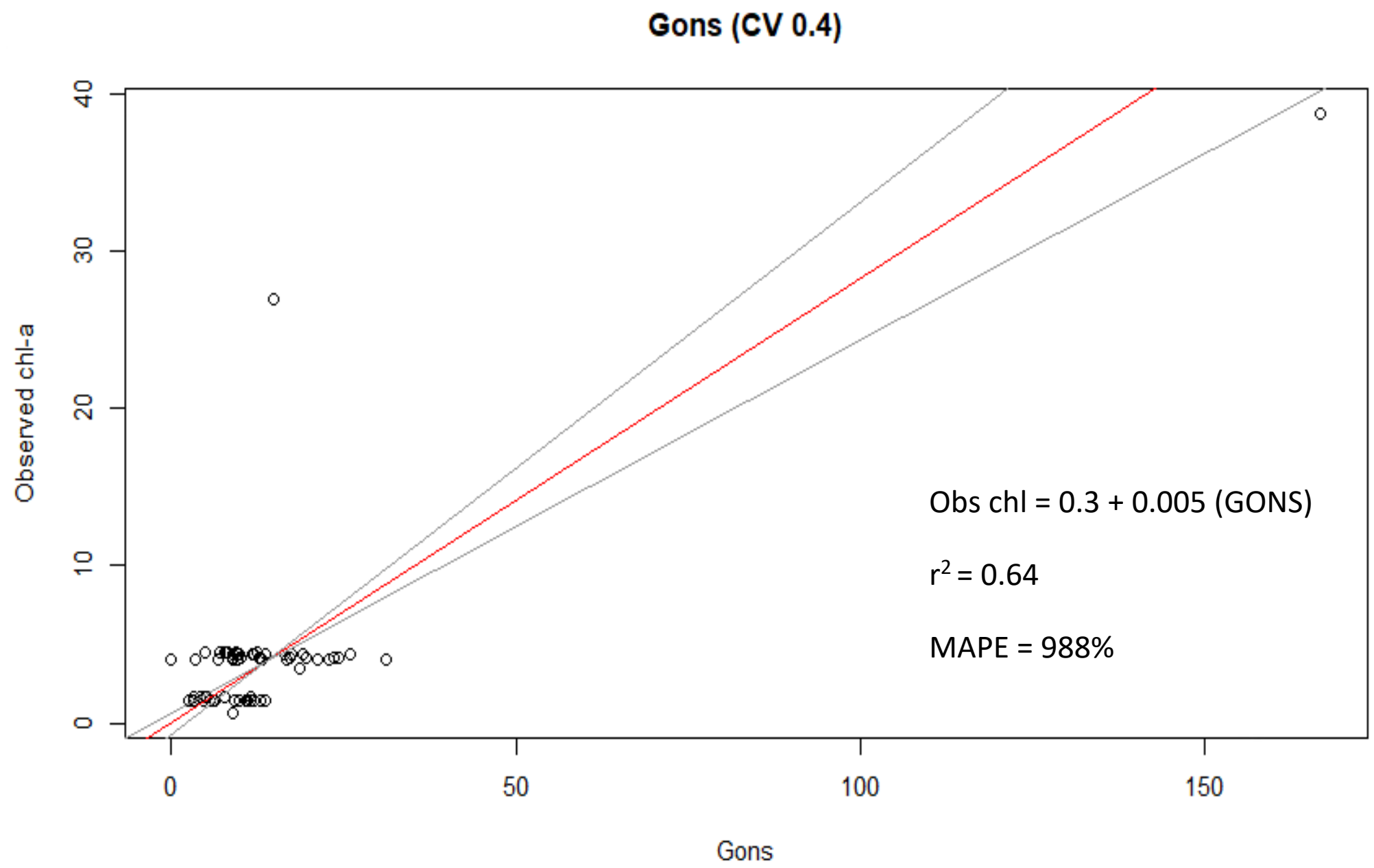

Figure S1. Scatterplot comparing observed chlorophyll-a and results from Gons [18] algorithm with the CV threshold at 0.4 (40%), and the corresponding statistical results. Data from Acolite atmospheric preprocessor.

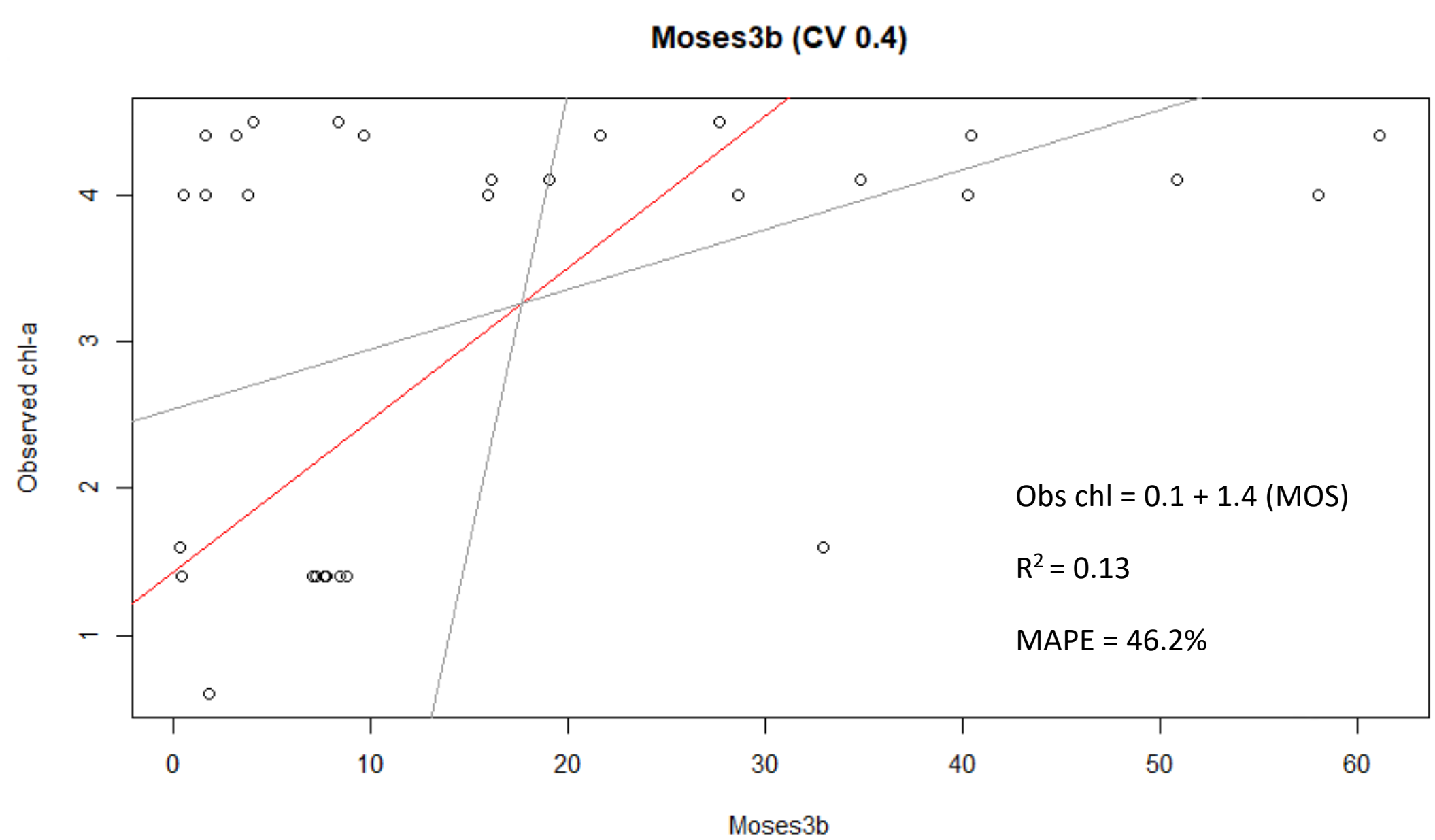

Figure S2. Scatterplot comparing observed chlorophyll-a and results from Moses3b [19] algorithm with the CV threshold at 0.4 (40%), and the corresponding statistical results. Data from Acolite atmospheric preprocessor.

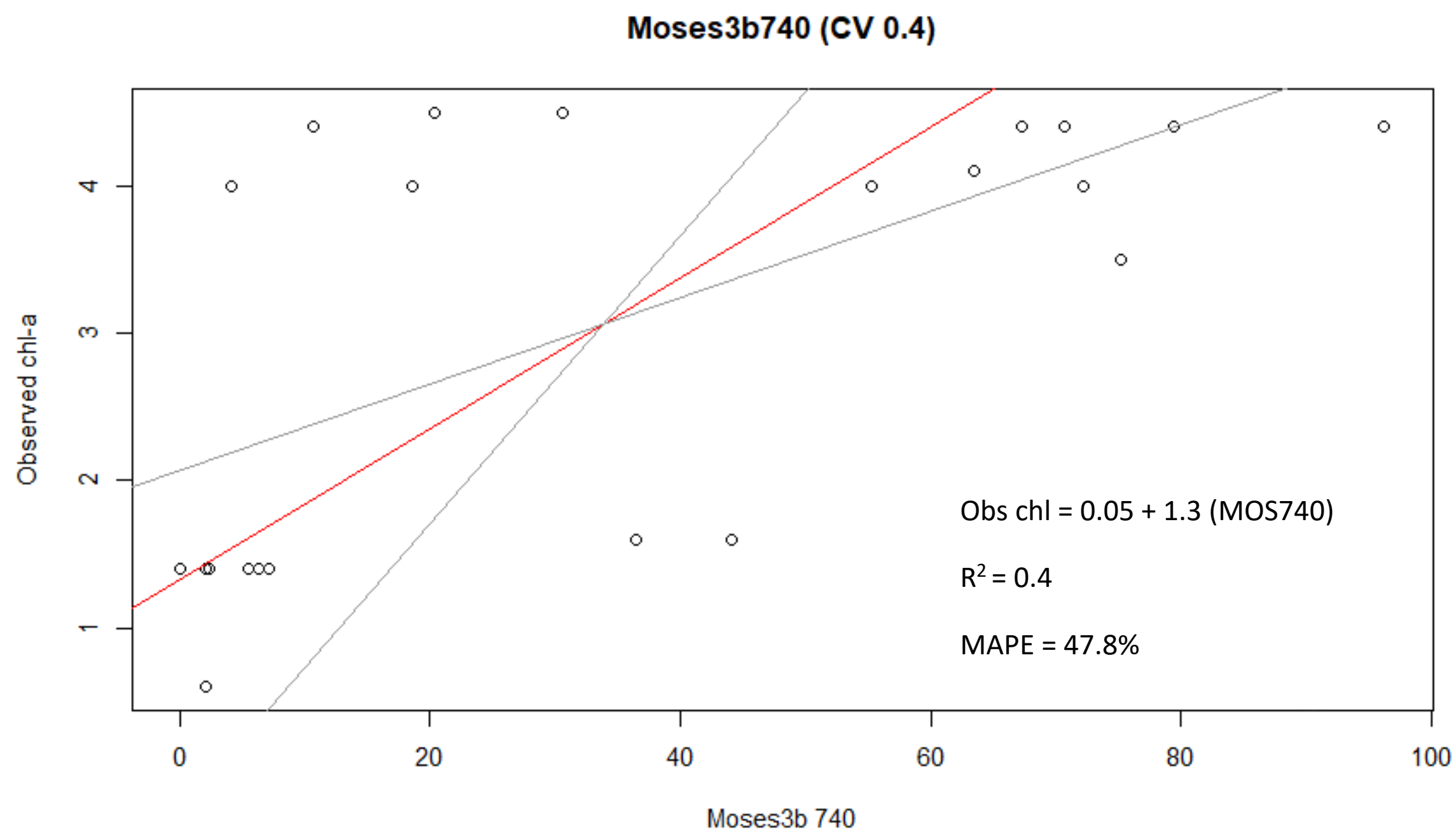

Figure S3. Scatterplot comparing observed chlorophyll-a and results from Moses3b 740 [19] algorithm with the CV threshold at 0.4 (40%), and the corresponding statistical results. Data from Acolite atmospheric preprocessor.

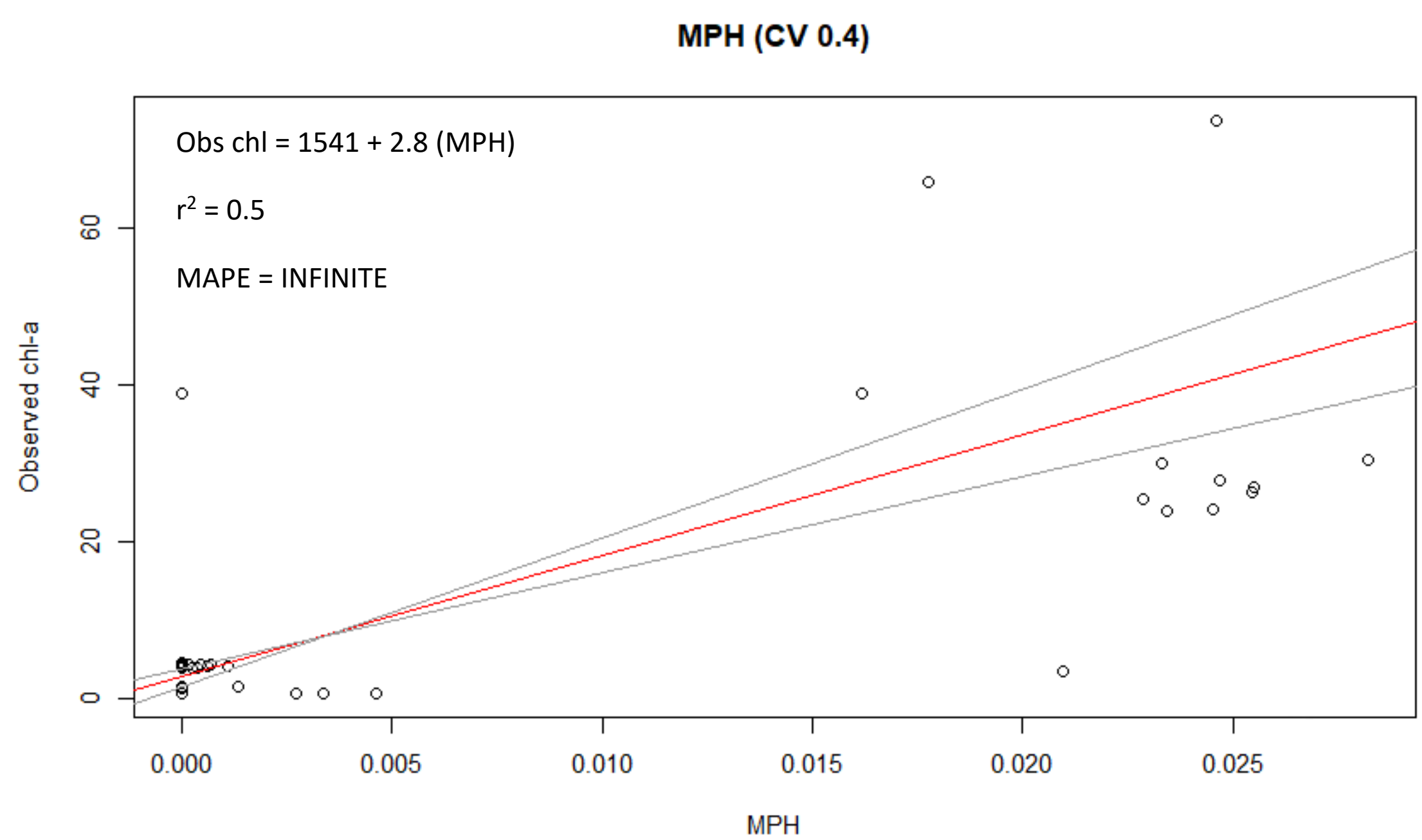

Figure S4. Scatterplot comparing observed chlorophyll-a and results from MPH [60] algorithm with the CV threshold at 0.4 (40%), and the corresponding statistical results. Data from Acolite atmospheric preprocessor.

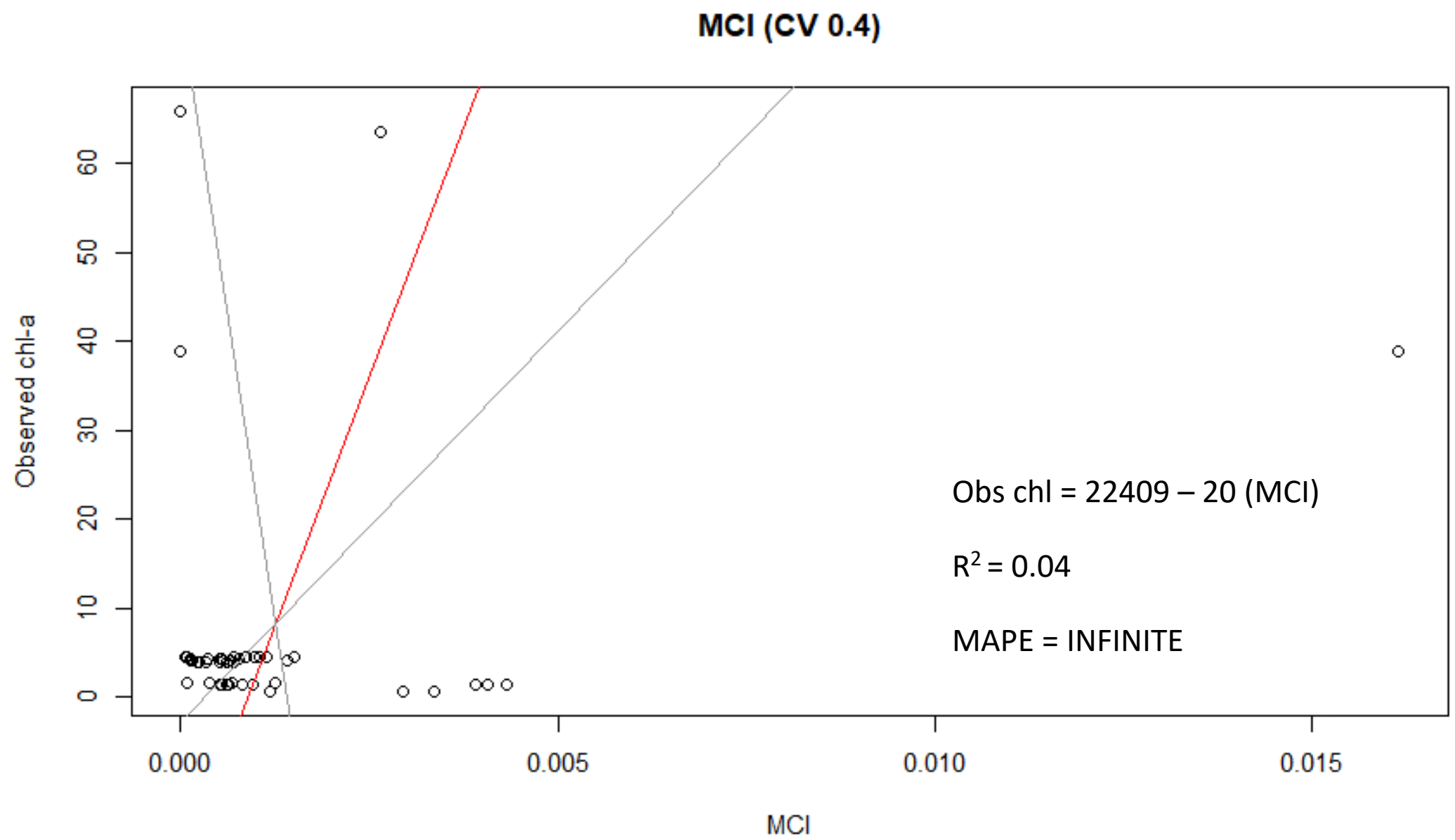

Figure S5. Scatterplot comparing observed chlorophyll-a and results from MCI [58] algorithm with the CV threshold at 0.4 (40%), and the corresponding statistical results. Data from Acolite atmospheric preprocessor.

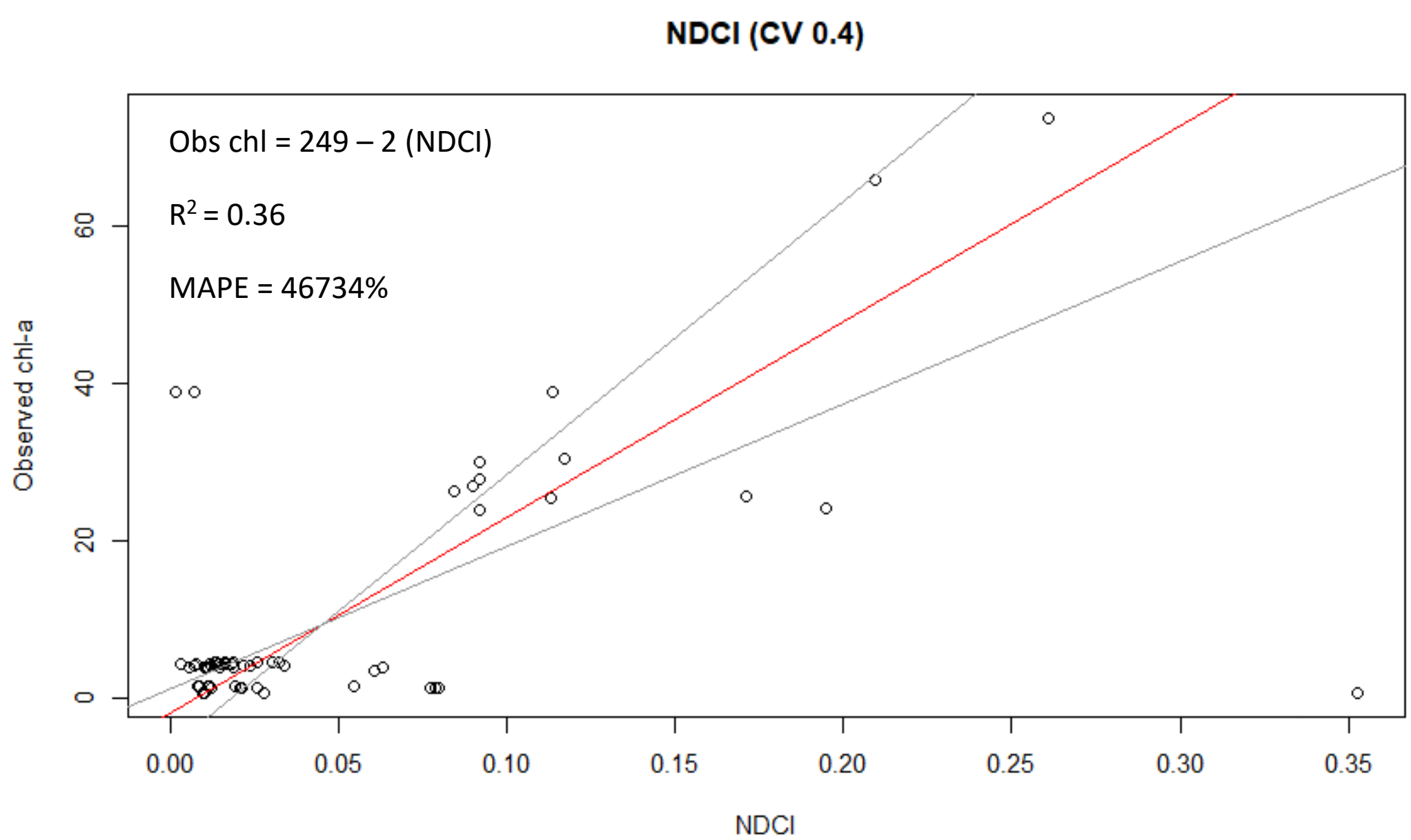

Figure S6. Scatterplot comparing observed chlorophyll-a and results from NDCI [56] algorithm with the CV threshold at 0.4 (40%), and the corresponding statistical results. Data from Acolite atmospheric preprocessor.

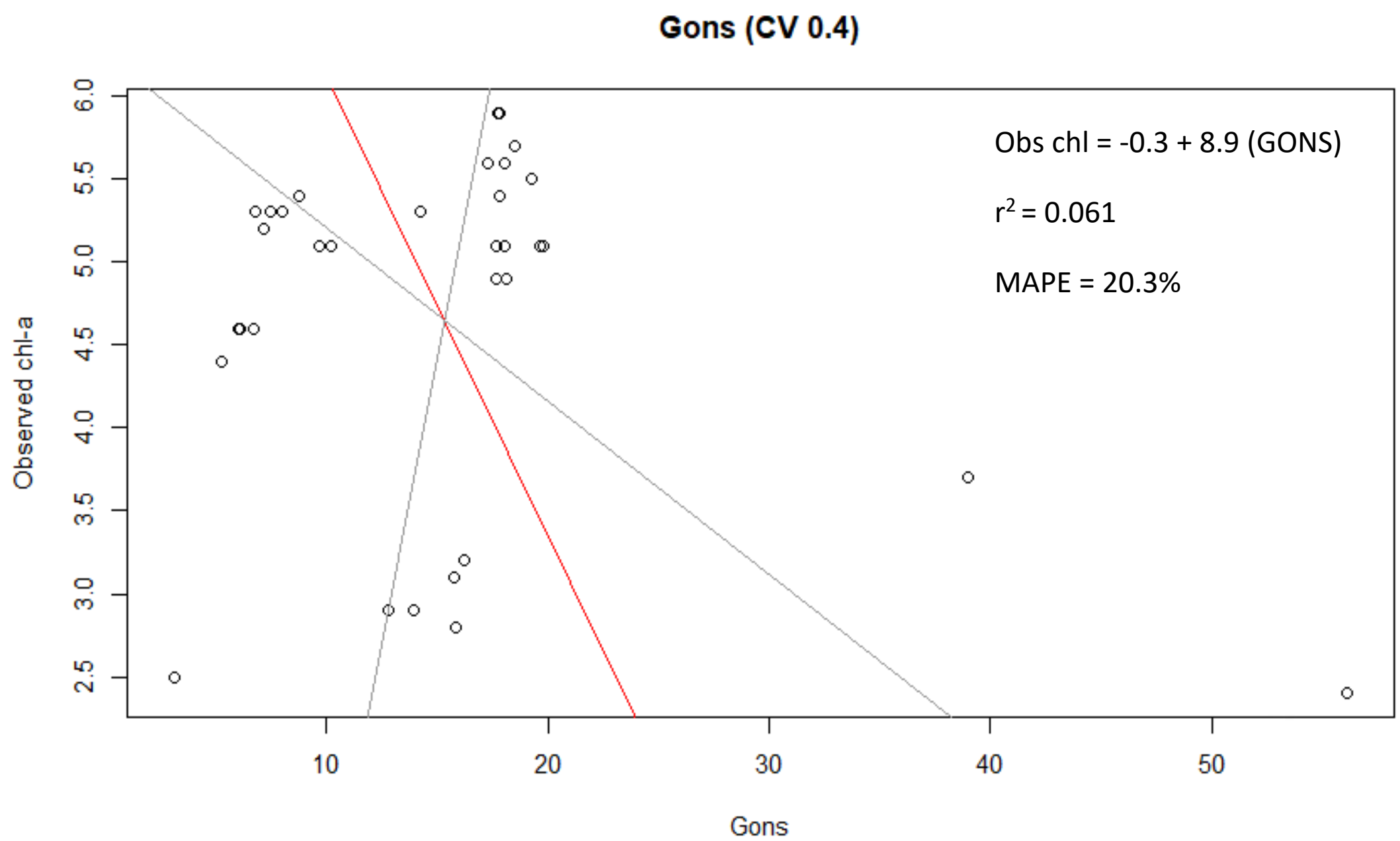

Figure S7. Scatterplot comparing observed chlorophyll-a and results from Gons [18] algorithm with the CV threshold at 0.4 (40%), and the corresponding statistical results. Data from Polymer atmospheric preprocessor.

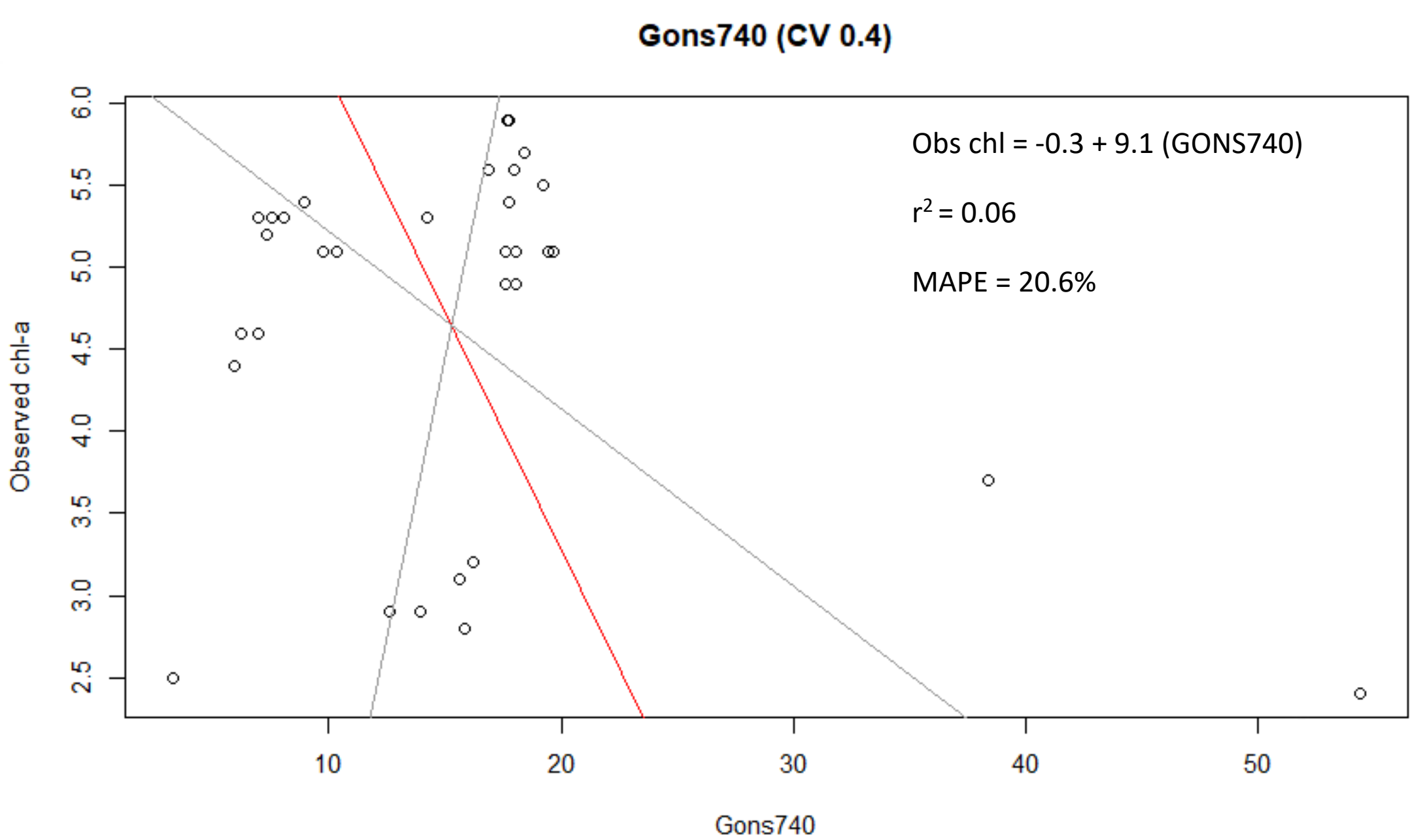

Figure S8. Scatterplot comparing observed chlorophyll-a and results from Gons 740 [18] algorithm with the CV threshold at 0.4 (40%), and the corresponding statistical results. Data from Polymer atmospheric preprocessor.

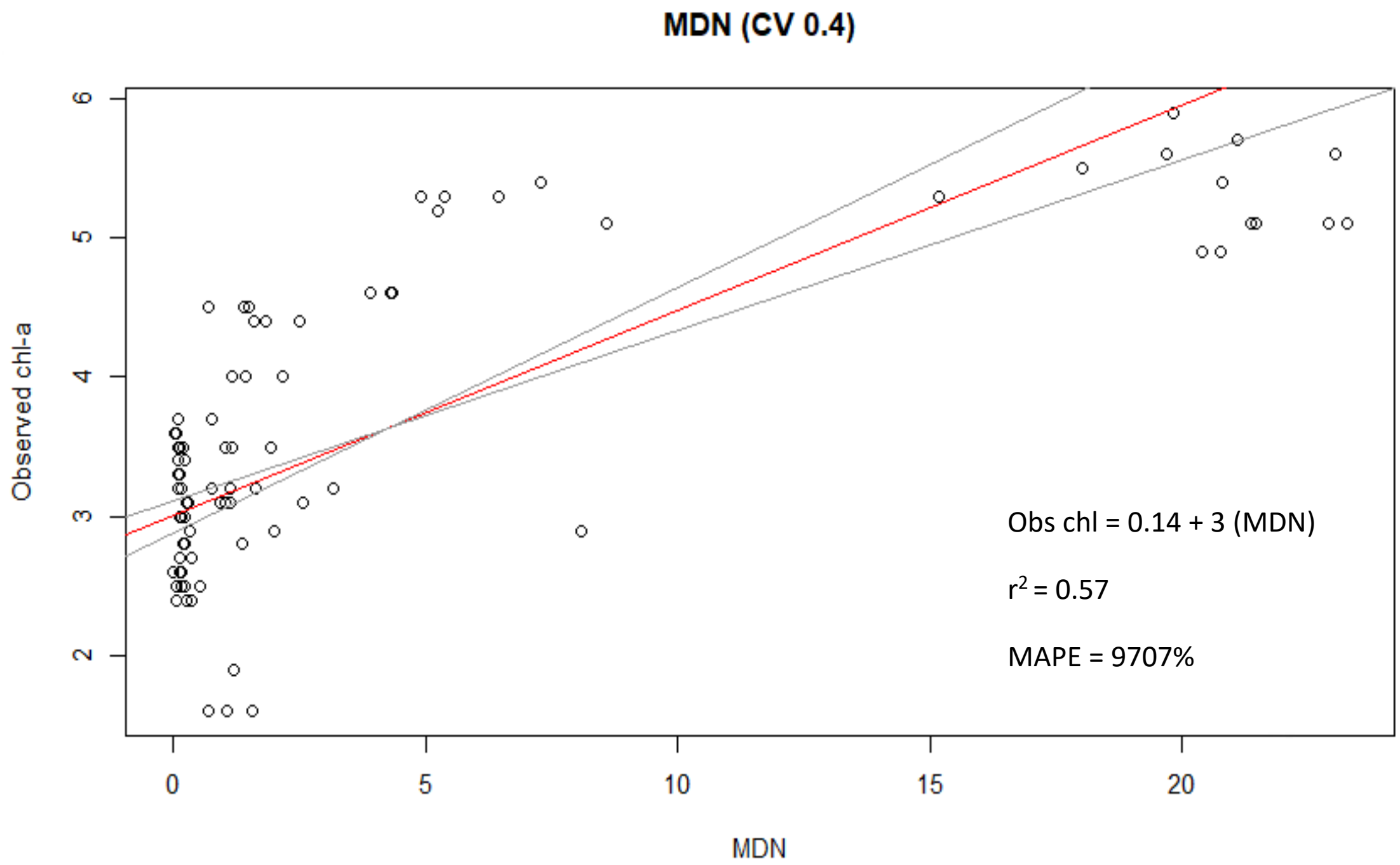

Figure S9. Scatterplot comparing observed chlorophyll-a and results from MDN [55] algorithm with the CV threshold at 0.4 (40%), and the corresponding statistical results. Data from Polymer atmospheric preprocessor.

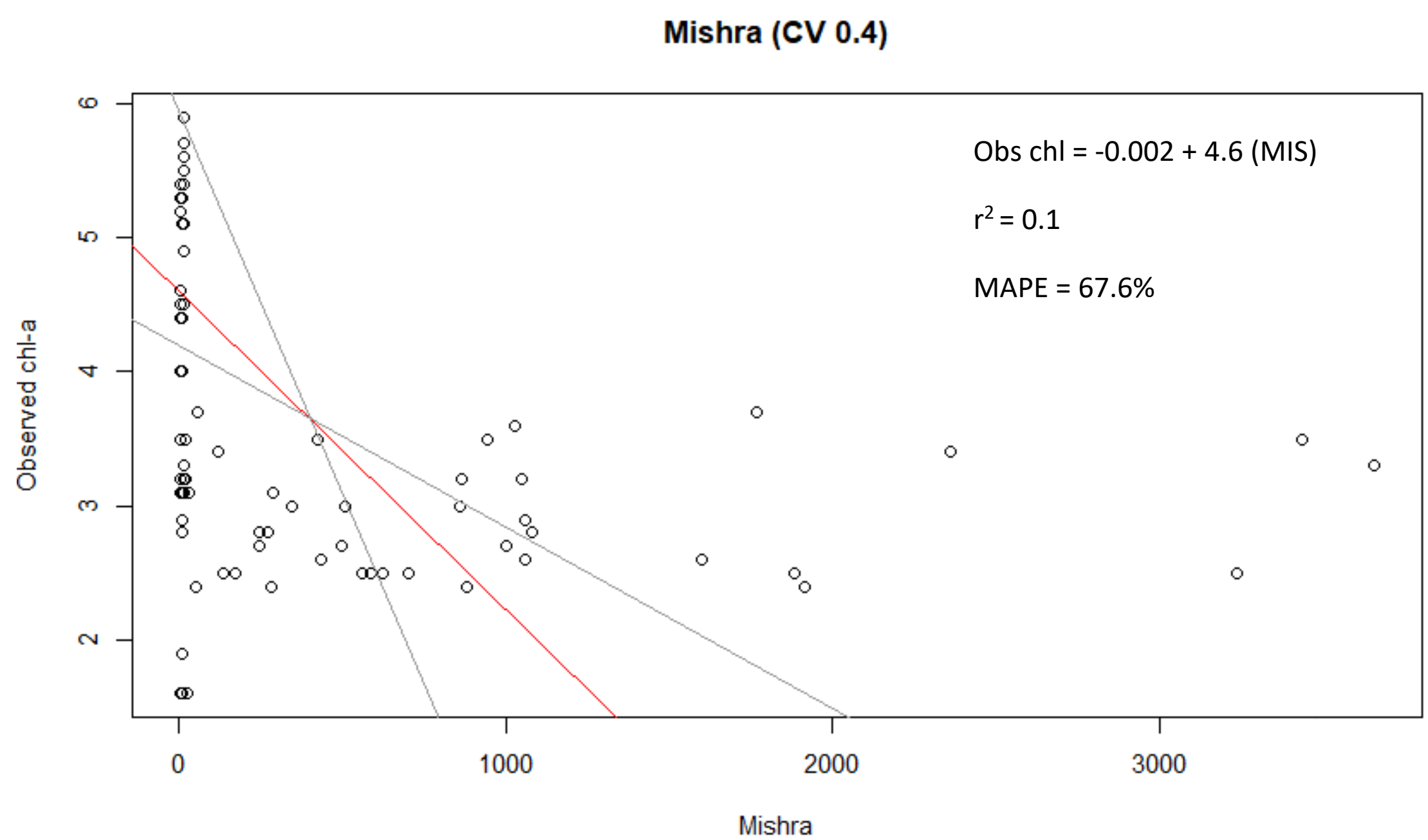

Figure S10. Scatterplot comparing observed chlorophyll-a and results from Mishra [58] algorithm with the CV threshold at 0.4 (40%), and the corresponding statistical results. Data from Polymer atmospheric preprocessor.

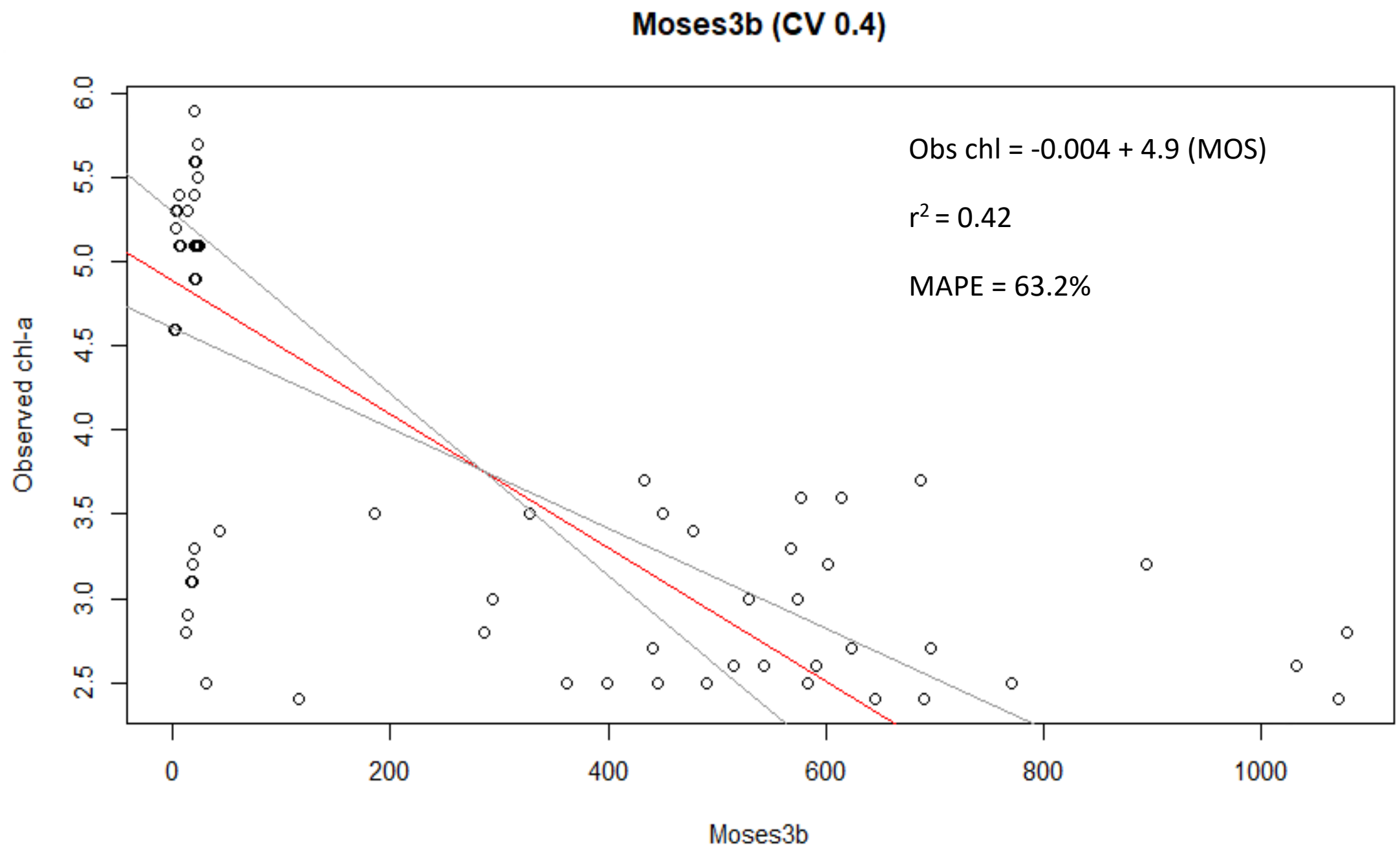

Figure S11. Scatterplot comparing observed chlorophyll-a and results from Moses3b [19] algorithm with the CV threshold at 0.4 (40%), and the corresponding statistical results. Data from Polymer atmospheric preprocessor.

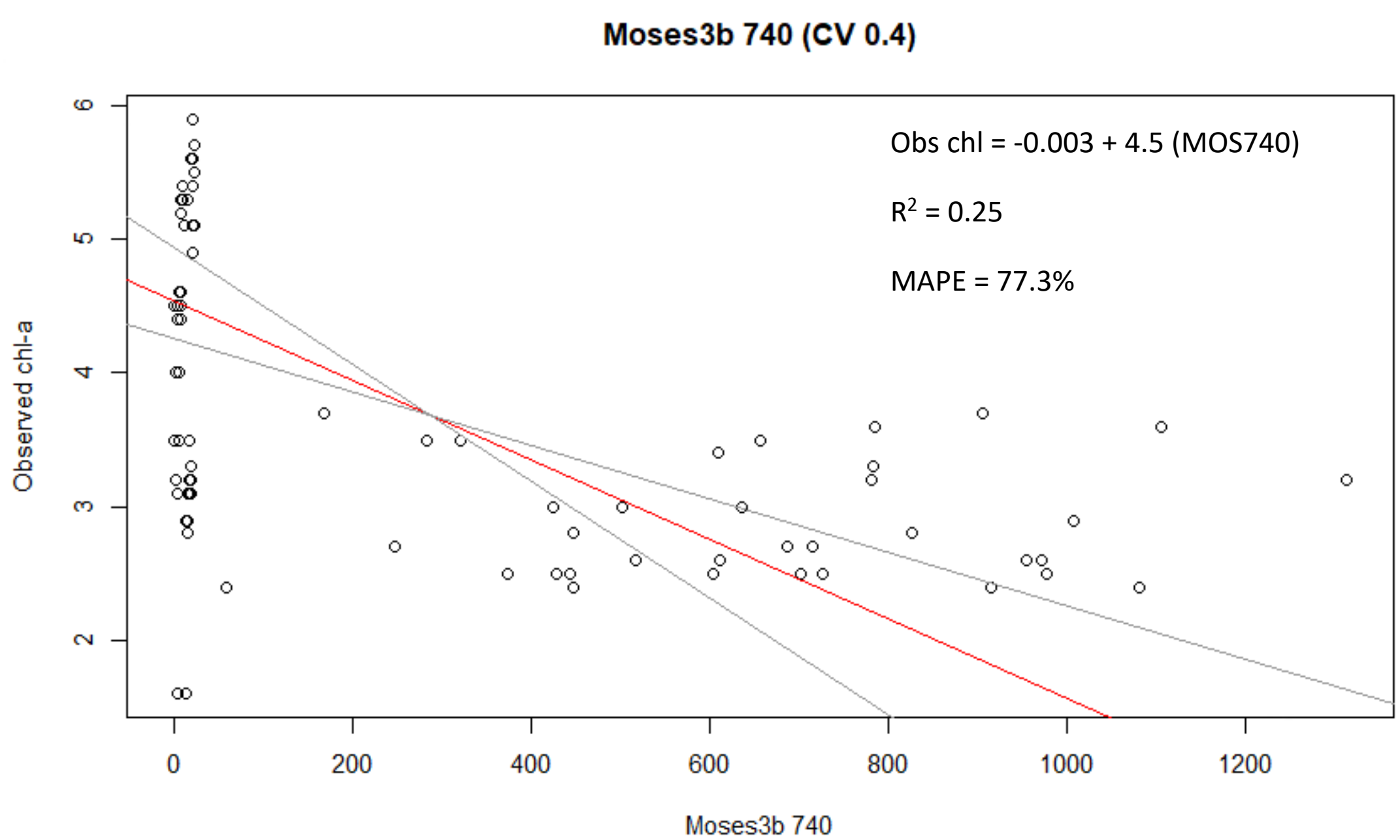

Figure S12. Scatterplot comparing observed chlorophyll-a and results from Moses3b 740 [19] algorithm with the CV threshold at 0.4 (40%), and the corresponding statistical results. Data from Polymer atmospheric preprocessor.

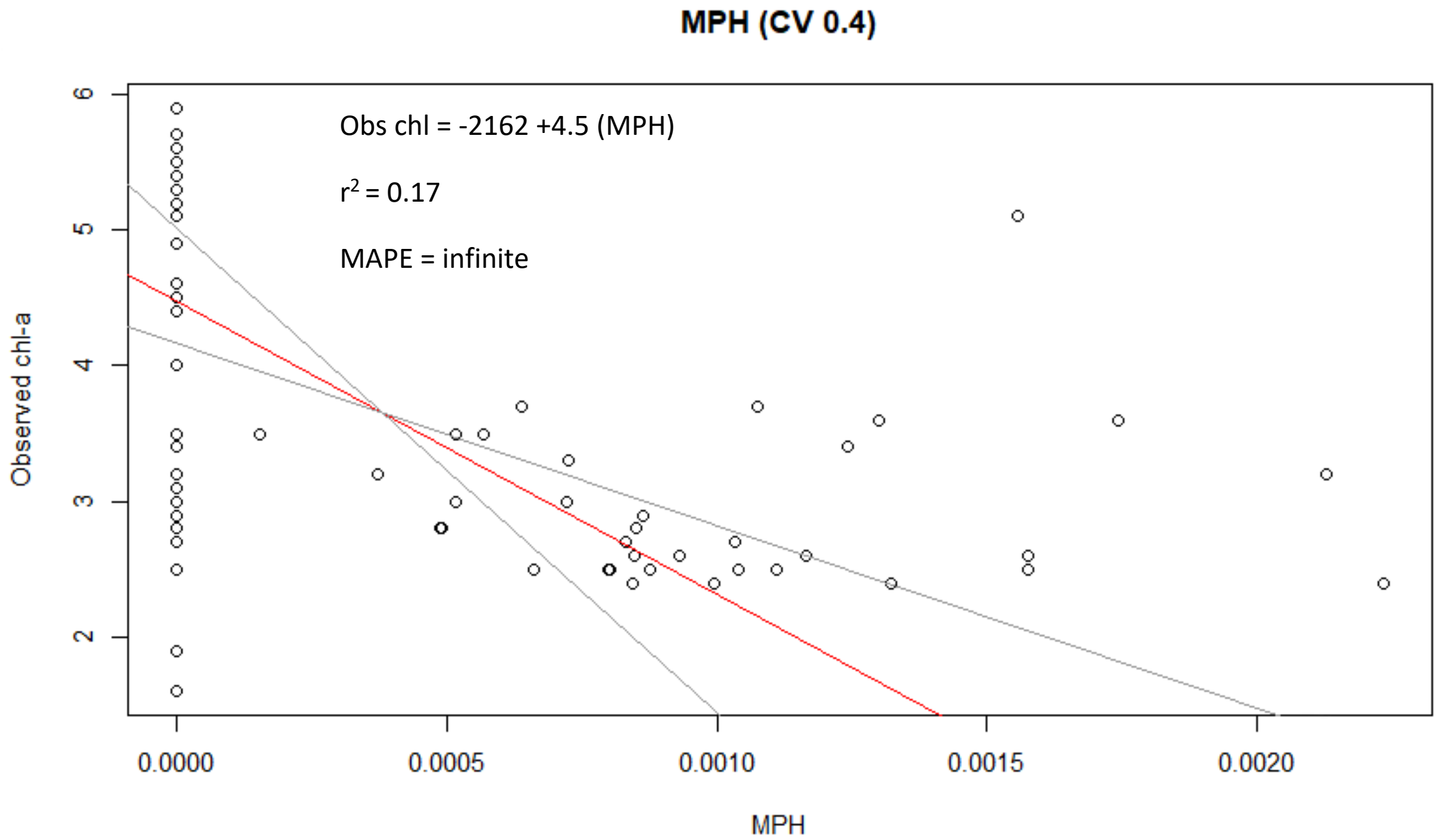

Figure S13. Scatterplot comparing observed chlorophyll-a and results from MPH [59] algorithm with the CV threshold at 0.4 (40%), and the corresponding statistical results. Data from Polymer atmospheric preprocessor.

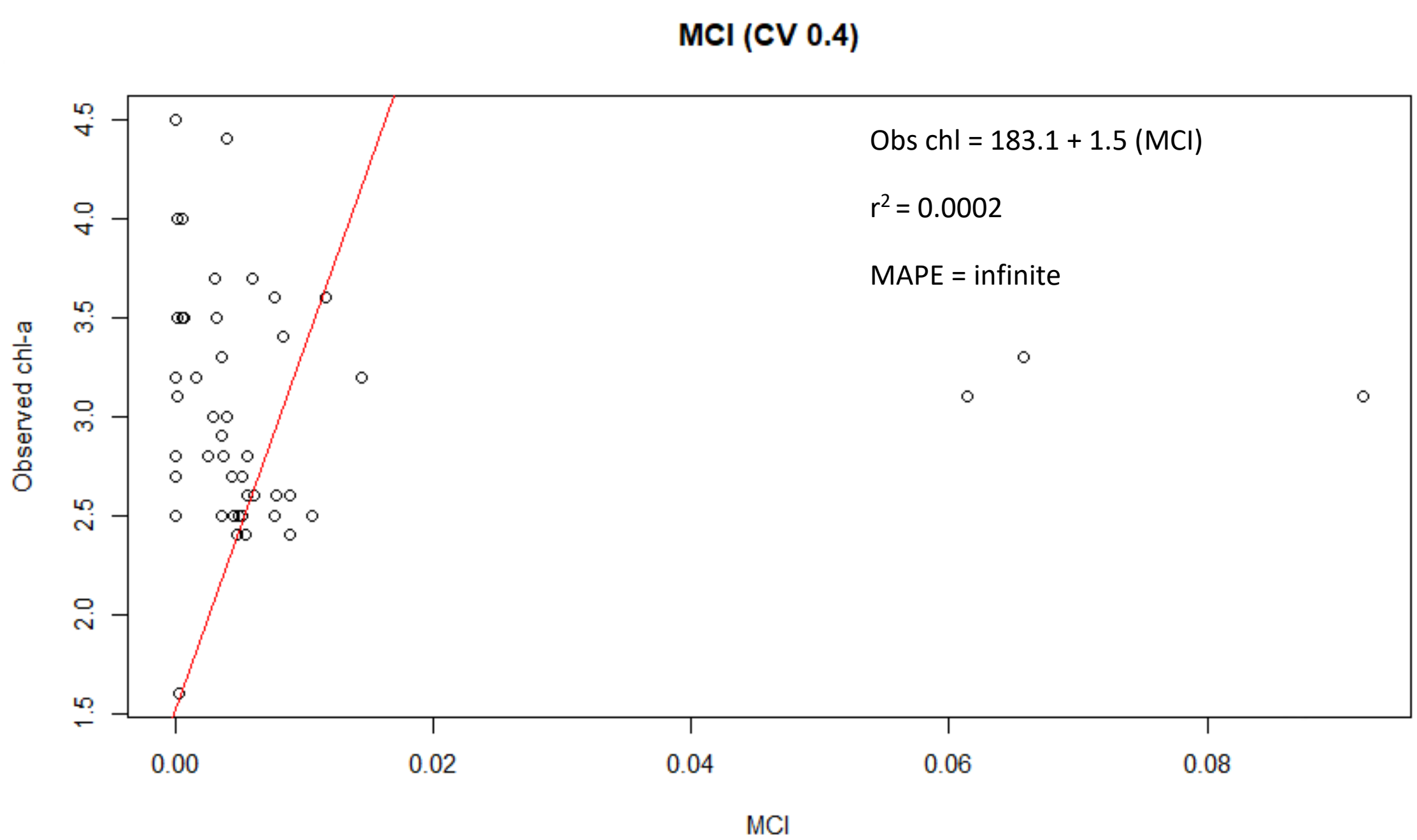

Figure S14. Scatterplot comparing observed chlorophyll-a and results from MCI [58] algorithm with the CV threshold at 0.4 (40%), and the corresponding statistical results. Data from Polymer atmospheric preprocessor.

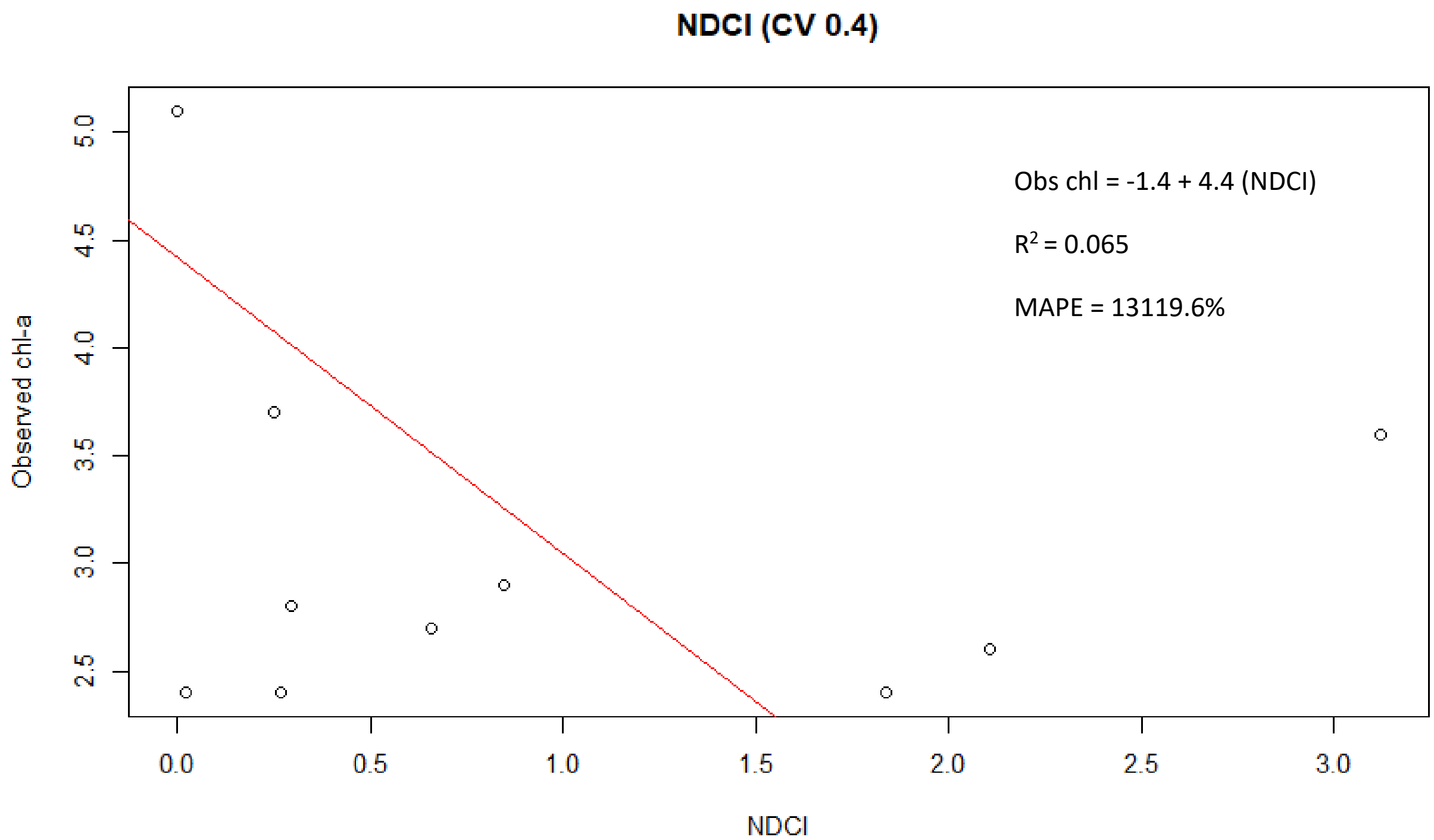

Figure S15. Scatterplot comparing observed chlorophyll-a and results from NDCI [56] algorithm with the CV threshold at 0.4 (40%), and the corresponding statistical results. Data from Polymer atmospheric preprocessor.

Table S1. Fitting statistics for regressions of observed versus estimated chlorophyll based on various algorithms. Data processed with Acolite AC. CV = 15% for QC filtering. CI = confidence interval, sterr = standard error, MAE_{mult} = multiplicative mean absolute error, MAPE = mean absolute percentage error, MB = multiplicative bias.

Statistic	Gons	Gons740	MDN	Mishra	Moses3b	Moses3b 740	MCI	MPH	NDCI
Reference	[18]	[18]	[55]	[57]	[19]	[19]	[58]	[59]	[56]
n	71	74	80	76	30	24	47	76	61
Intercept	-499.7	-119.2	-9.5	-2.9	1.4	1.3	5.9	34.5	16.2
Slope	44.5	11.6	4	0.7	0.1	0.05	2090	-186300	-468.7
Pval	0.3	0.23	0.05	0.002	0.02	0.001	0.4	0.034	0.001
CI2.5_int	105.8	91.6	-64.5	-12.4	-7.6	-0.33	NA	15	7.8
CI97.5_int	-61.3	-22.2	3.9	-0.42	2.5	2	NA	-10.2	-11.1
CI2.5_slope	6.1	2.77	0.7	0.4	0.04	0.03	NA	123469	579
CI97.5_slope	-8.5	-7.6	17.6	1.8	0.61	0.1	NA	-50460	-145.5
R-squared	0.002	0.009	0.04	0.11	0.13	0.4	0.001	0.009	0.02
Average	8.35	8.16	6.8	2.92	3.26	3.05	8.03	7.62	4
Med	4	4	4	4	4	4	4	4	4
sterr	2.2	2.1	1.7	0.16	0.25	0.3	2.5	1.9	0.85
MeAbsEr	1.02	1.02	1	1.02	1.02	1.01	1.1	Infinite	1
MeAbsPerEr	1103.6	153.5	301.4	57.5	48.2	49.5	1022739	Infinite	47345.1
MeAbsBi	9.2	9.2	3.1	6.75	7.62	2.4	0.0006	0	0.35

Table S2. Fitting statistics for regressions of observed versus estimated chlorophyll based on various algorithms. Data processed with Acolite AC. CV = 20% for QC filtering. CI = confidence interval, sterr = standard error, MAE_{mult} = multiplicative mean absolute error, MAPE = mean absolute percentage error, MB = multiplicative bias.

Statistic	Gons	Gons740	MDN	Mishra	Moses3b	Moses3b 740	MCI	MPH	NDCI
Reference	[18]	[18]	[55]	[57]	[19]	[19]	[58]	[59]	[56]
n	80	83	91	95	38	24	48	80	70
intercept	-82.1	-23.7	-25.4	-21.8	-148.3	1.3	4.3	-8.2	-17.2
slope	8.2	2.7	6.5	3.1	9	0.05	3682	58633	768.2
pval	0.2	0.06	0.1	0.001	0.4	0.002	0.4	0.1	0.1
CI2.5_int	71.3	-160	270.4	-34.2	NA	-0.33	NA	99.2	51.2
CI97.5_int	-6.9	-0.65	-7.7	-12.8	NA	2.04	NA	1	-3.3
CI2.5_slope	1.4	0.7	3	2.2	NA	0.03	NA	25701	297
CI97.5_slope	-5.7	14.6	-53.3	4.4	NA	0.1	NA	-327583	-1556.5
R-squared	0.01	0.04	0.033	0.26	0.0003	0.4	0.0005	0.03	0.03
Average	8.15	7.1	6.7	8.2	9.8	3.05	8	8.1	5.4
Med	4	4	4	4	4.05	4	4	4	4
sterr	1.9	1.7	1.5	1.7	3.1	0.3	2.5	2	1.02
MeAbsEr	1.02	1.02	1.01	1.01	1.02	1.01	1.1	Infinite	1
MeAbsPerEr	1033	121.2	271.4	75.8	58.2	44.7	873834	Infinite	43652
MeAbsBi	9.2	9.2	3.1	6.7	7.6	2.4	0.0006	0	0.35

Table S3. Fitting statistics for regressions of observed versus estimated chlorophyll based on various algorithms. Data processed with Polymer AC. CV = 15% for QC filtering. CI = confidence interval, sterr = standard error, MAE_{mult} = multiplicative mean absolute error, MAPE = mean absolute percentage error, MB = multiplicative bias.

Statistic	Gons	Gons740	MDN	Mishra	Moses3b	Moses3b 740	MCI	MPH	NDCI
Reference	[18]	[18]	[55]	[57]	[19]	[19]	[58]	[59]	[56]
n	24	24	62	60	39	56	28	61	7
intercept	6.8	6.8	3	4.6	5	4.5	4.8	4.4	4.8
slope	-0.14	-0.13	0.16	-0.004	-0.004	-0.003	-284.5	-2494.5	-1.6
pval	0.2	0.2	0.001	0.001	0.001	0.001	0.46	0.003	0.4
CI2.5_int	NA	NA	2.8	4.3	4.8	4.3	3.1	4.1	NA
CI97.5_int	NA	NA	3.2	5.4	5.3	4.9	2.9	5.1	NA
CI2.5_slope	NA	NA	0.12	-0.0086	-0.0048	-0.0054	18.4	-4912.7	NA
CI97.5_slope	NA	NA	0.2	-0.0025	-0.003	-0.002	-10	-1210.7	NA
R-squared	0.03	0.03	0.5	0.2	0.62	0.26	0.0023	0.14	0.036
Average	4.9	4.9	3.8	3.8	4	3.8	3.1	3.8	3.6
Med	5.1	5.1	3.55	3.6	4.6	3.65	3	3.6	3.6
sterr	0.15	0.15	0.15	0.15	0.2	0.16	0.13	0.15	0.42
MAE	1.04	1.04	1.02	1.04	1.08	1.06	1.12	1.1	1.04
MAPE	20.28	20.65	548.8	57.03	53.75	75.9	Infinite	Infinite	19448
MB	38.16	37.6	0.75	53.2	424.9	165	0.003	0.0006	0.25

Table S4. Fitting statistics for regressions of observed versus estimated chlorophyll based on various algorithms. Data processed with Polymer AC. CV = 20% for QC filtering. CI = confidence interval, sterr = standard error, MAE_{mult} = multiplicative mean absolute error, MAPE = mean absolute percentage error, MB = multiplicative bias.

Statistic	Gons	Gons740	MDN	Mishra	Moses3b	Moses3b 740	MCI	MPH	NDCI
Reference	[18]	[18]	[55]	[57]	[19]	[19]	[58]	[59]	[56]
n	32	32	86	85	61	77	44	86	11
Intercept	8.4	8.6	3	4.6	4.9	4.5	6.6	4.5	4.4
Slope	-0.25	-0.26	0.15	-0.003	-0.004	-0.003	-604	-2360.5	-1.37
Pval	0.082	0.1	0.001	0.001	0.001	0.001	0.51	0.001	0.26
CI2.5_int	6.1	6.1	2.8	4.2	4.6	4.2	3.3	4.2	NA
CI97.5_int	-7	-6.9	3.1	5.9	5.3	4.8	2.6	5.1	NA
CI2.5_slope	0.8	0.75	0.12	-0.006	-0.0056	-0.004	70.2	-3964.1	NA
CI97.5_slope	-0.1	-0.1	0.18	-0.0016	-0.003	-0.002	-54.3	-1477.5	NA
R-squared	0.07	0.07	0.55	0.11	0.45	0.28	0.0012	0.17	0.065
Average	4.6	4.6	3.6	3.6	3.8	3.7	3	3.6	3.2
Med	5.1	5.1	3.4	3.4	3.4	3.5	2.8	3.4	2.8
sterr	0.18	0.18	0.12	0.12	0.15	0.13	0.09	0.12	0.31
MAE	1.02	1.02	1.01	1.03	1.05	1.04	1.08	1.1	1.03
MAPE	20.9	21.2	10230.6	66.5	61.4	76.5	infinite	infinite	14004
MB	38.4	37.8	0.75	53.5	427.3	166	0.003	0.00063	0.25

References

18. Gons, H.J. A Chlorophyll-Retrieval Algorithm for Satellite Imagery (Medium Resolution Imaging Spectrometer) of Inland and Coastal Waters. *J. Plankton Res.* **2002**, *24*, 947–951. https://doi.org/10.1093/plankt/24.9.947.

19. Moses, W.J.; Gitelson, A.A.; Berdnikov, S.; Saprygin, V.; Povazhnyi, V. Operational MERIS-Based NIR-Red Algorithms for Estimating Chlorophyll-a Concentrations in Coastal Waters—The Azov Sea Case Study. *Remote Sens. Environ.* **2012**, *121*, 118–124. https://doi.org/10.1016/j.rse.2012.01.024.

55.. Pahlevan, N.; Smith, B.; Schalles, J.; Binding, C.; Cao, Z.; Ma, R.; Alikas, K.; Kangro, K.; Gurlin, D.; Hà, N.; et al. Seamless Retrievals of Chlorophyll-a from Sentinel-2 (MSI) and Sentinel-3 (OLCI) in Inland and Coastal Waters: A Machine-Learning Approach. *Remote Sens. Environ.* **2020**, *240*, 111604. https://doi.org/10.1016/j.rse.2019.111604.

56.. Mishra, S.; Mishra, D.R. Normalized Difference Chlorophyll Index: A Novel Model for Remote Estimation of Chlorophyll-a Concentration in Turbid Productive Waters. *Remote Sens. Environ.* **2012**, *117*, 394–406. https://doi.org/10.1016/j.rse.2011.10.016.

57. Mishra, S.; Stumpf, R.P.; Schaeffer, B.; Werdell, P.J.; Loftin, K.A.; Meredith, A. Evaluation of a Satellite-Based Cyanobacteria Bloom Detection Algorithm Using Field-Measured Microcystin Data. *Sci. Total Environ.* **2021**, *774*, 145462. https://doi.org/10.1016/j.scitotenv.2021.145462.

58. Salls, W.B.; Schaeffer, B.A.; Pahlevan, N.; Coffer, M.M.; Seegers, B.N.; Werdell, P.J.; Ferriby, H.; Stumpf, R.P.; Binding, C.E.; Keith, D.J. Expanding the Application of Sentinel-2 Chlorophyll Monitoring across United States Lakes. *Remote Sens.* **2024**, *16*, 1977. https://doi.org/10.3390/rs16111977.

59. Matthews, M.W.; Bernard, S.; Robertson, L. An Algorithm for Detecting Trophic Status (Chlorophyll-a), Cyanobacterial-Dominance, Surface Scums and Floating Vegetation in Inland and Coastal Waters. *Remote Sens. Environ.* **2012**, *124*, 637–652. https://doi.org/10.1016/j.rse.2012.05.032.
